# Efficacy of Chlorhexidine after Oral Surgery Procedures on Wound Healing: Systematic Review and Meta-Analysis

**DOI:** 10.3390/antibiotics12101552

**Published:** 2023-10-20

**Authors:** María de Nuria Romero-Olid, Elena Bucataru, Pablo Ramos-García, Miguel Ángel González-Moles

**Affiliations:** 1 School of Dentistry, University of Granada, 18071 Granada, Spain; nromero@ugr.es (M.d.N.R.-O.); elenabu@correo.ugr.es (E.B.); magonzal@ugr.es (M.Á.G.-M.); 2 Instituto de Investigación Biosanitaria ibs.GRANADA, 18012 Granada, Spain

**Keywords:** chlorhexidine, wound healing, oral surgery, systematic review and meta-analysis

## Abstract

Our objective was to evaluate qualitatively and quantitatively, through a systematic review and meta-analysis, available evidence on the efficacy of chlorhexidine (CHX) when applied after oral surgery on wound healing and related clinical parameters. MEDLINE/PubMed, Embase, CENTRAL, Web of Science, and Scopus were searched for studies published before January 2023. The quality of the methodology used in primary-level studies was assessed using the RoB2 tool; meta-analyses were performed jointly with heterogeneity and small-study effect analyses. Thirty-three studies and 4766 cases were included. The results point out that the application of CHX was significantly more effective, compared to controls where CHX was not employed, providing better wound healing after oral surgery (RR = 0.66, 95% CI = 0.55–0.80, *p* < 0.001). Stratified meta-analyses confirmed the higher efficacy of 0.20% CHX gel vs. other vehicles and concentrations (*p* < 0.001, respectively). Likewise, the addition of chitosan to CHX significantly increased the efficacy of surgical wound healing (*p* < 0.001). The use of CHX has also been significantly beneficial in the prevention of alveolar osteitis after any type of dental extraction (RR = 0.46, 95% CI = 0.39–0.53, *p* < 0.001) and has also been effective when applied as a gel for a reduction in pain after the surgical extraction of third molars (MD = −0.97, 95% CI = −1.26 to −0.68, *p* < 0.001). In conclusion, this systematic review and meta-analysis demonstrate on the basis of evidence that the application of CHX exerts a beneficial effect on wound healing after oral surgical procedures, significantly decreasing the patient’s risk of developing surgical complications and/or poor wound healing. This benefit was greater when CHX was used at 0.20% in gel form with the addition of chitosan.

## 1. Introduction

Wound healing in oral surgery is a complex and dynamic process that culminates in the restitution of the tissue’s integrity [1,2]. During the healing process, a series of successive events occurred, starting with local hemostasis, followed by inflammation, tissue proliferation (mainly of fibroblasts, epithelial and endothelial cells), neoangiogenesis, repithelialization, synthesis, union and the alignment of collagen fibers, formation of granulation tissue, and definitive tissue remodeling [2,3,4]. These events can be altered by several factors, such as diseases (e.g., diabetes mellitus [5], hypothyroidism [6,7], or rheumatoid arthritis [8]), drug intake (e.g., bisphosphonates [7,9]), tobacco use [10]) or infections as a consequence of bacterial imbalance in the wound site [11,12,13]. Consequently, patients undergoing oral surgery are at an increased risk of developing complications during postoperative wound healing [14,15], such as delayed or the absence of healing, pain and, particularly, after the extraction of third molars, the development of alveolar osteitis [16,17,18,19,20]. The establishment of improvement strategies to minimize wound healing complications after oral surgery is currently a priority line of clinical research in modern dentistry [21,22].

One of the strategies that has received the most attention is the application of chlorhexidine (CHX) to wounds after oral surgical procedures [23]. This approach is justified by the broad-spectrum actions of CHX against Gram-positive and Gram-negative bacteria, with bacteriostatic properties at low doses and bactericidal properties at high concentrations [24,25], as well as an antifungal effect [23]. Its mechanism of action is exerted by increasing the permeability of the cell membrane of target microorganisms, which causes the precipitation of macromolecules in the cytoplasm and subsequent microbial death via cell lysis [23]. Therefore, the effect of CHX is mainly based on bacterial load reduction, playing an anti-infection role, which seems essential in the early stages of wound healing. Nevertheless, the problem of healing is more complicated, in which many more factors are involved.

In recent years, the use of CHX has increased exponentially in the different fields of medicine, particularly in dentistry, where it is currently the most widely used antiseptic due to its antimicrobial action and other additional advantages for clinical practice and for the patient, such as its low cost and ease of application [23]. CHX can also be presented in different vehicles (mouthwash, gel, spray, toothpaste, etc.) and in variable concentrations (mainly 0.12% and 0.20%), which allows its use to be adapted to the patient’s needs [23]. Given the proven benefits of CHX, its application has been proposed to improve wound healing after oral surgical procedures [26]. However, it is surprising that, to date, there have been no scientific publications in the form of systematic reviews and meta-analyses offering a high level of evidence on the relevance of its indication.

In light of the above, we propose the present study with the objective of evaluating qualitatively and quantitatively, based on the evidence, through a systematic review and meta-analysis, the efficacy of the application of CHX after oral surgery on wound healing for the improvement of clinical parameters in relation to this biological process and on the possible complications that may occur, among which we can include epithelialization, a reduction in erythema, suture dehiscence, the development of alveolar osteitis and pain.

## 2. Materials and Methods

The present study was conducted closely by rigorously following *Cochrane Collaboration* criteria for systematic reviews of interventions [27]. The manuscript was prepared closely in compliance with the updated PRISMA statement (Preferred Reporting Items for Systematic Reviews and Meta-Analyses) [28].

### 2.1. Protocol

A preliminary methodological protocol was designed *a priori*, which was later submitted to PROSPERO International prospective register of systematic reviews (ID465899/CRD42023465899 code was assigned), with the goal of minimizing the risk of bias by reinforcing the transparency, precision, and integrity of this research. The study protocol also adhered to specific PRISMA-P reporting guidelines [29].

### 2.2. Search Strategy

We searched Embase, MEDLINE/PubMed, Scopus, and Web of Sciences databases, as well as registered Cochrane Central Register of Controlled Trials (CENTRAL), for primary-level studies published before the upper limit of January 2023 without lower date filters or limits. Electronic searches were driven by combining the thesaurus with free terms, which were designed in order to maximize sensitivity (Supplementary Information [S] Table S1). Furthermore, we conducted an extra screening process by manually searching through the reference lists of the retrieved studies and utilizing Google Scholar. All references were managed using Mendeley v.1.19.8 (Elsevier, Amsterdam, The Netherlands); the duplicates’ removal process was also driven using this software.

All references were organized and managed using Mendeley v.1.19.8 (Elsevier, Amsterdam, The Netherlands), and any duplicate references were removed via this software.

### 2.3. Eligibility Criteria

We formulated the subsequent PICO question: “Is the application of CHX effective in patients undergoing oral surgery procedures, compared to controls not exposed to CHX or other drugs, in order to improve wound healing and related clinical parameters (i.e., epithelization, erythema, wound dehiscence, alveolar osteitis, and pain)?”. Primary-level studies were strictly included according to the following eligibility criteria.

Inclusion criteria: Randomized clinical trials (RCTs) or quasi-RCTs (q-RCTs), with parallel groups or split-mouth design, were used without restrictions for publication language or date, geographical area, age or sex; CHX either alone or in association with other antiseptics or antibiotics was applied as experimental intervention and placebo or no-treatment as the control arm; evaluation of the risk of wound healing complications after oral surgery procedures was conducted in study groups. When two research arms were investigated by applying CHX in different concentrations or vehicles, both were included and considered as separate analysis units.

Exclusion criteria were as follows: retracted articles, non-randomized clinical trials or observational studies, case reports, preclinical experiments (animal experimentation or in vitro research), articles without scientific method and/or results (letters, editorials, personal opinions, commentaries, meeting abstracts, literature narrative reviews, or book chapters), as well as secondary/tertiary-evidence level studies (scoping reviews, systematic reviews with or without meta-analysis, overviews of reviews, umbrella studies, etc.); surgical procedures from anatomic areas distinct to the oral cavity; primary-level studies without control groups, or controls exposed to an experimental intervention with known antiseptic effect (e.g., CHX, antibiotics or other drugs); no analysis of clinical outcomes of interest or a lack of essential data for the statistical estimation of effect size metrics with their corresponding confidence intervals; inter-study overlapping populations.

### 2.4. Study Selection Process

Two blinded authors (EB and MNRO) independently applied eligibility criteria, later resolving any discrepancies via consensus with a third supervisor author (PRG). The records were selected across two subsequent stages. In stage I, titles and abstracts were screened, looking for potential records meeting inclusion criteria. In stage II, the records were read in full text and excluded if eligibility criteria were not met. Initially, all systematic reviewers underwent training and calibration rounds by piloting 80 random papers in order to become proficient in the process of identifying and selecting studies. An optimal inter-agreement proportional score (relative frequency of agreement = 98.75%) was obtained. The inter-rater reliability was also measured by calculating Cohen’s kappa statistic and obtaining an almost perfect agreement (κ = 0.90).

### 2.5. Data Extraction

Two authors (EB and PRG) systematically extracted data from included primary-level studies by employing standardized data collection forms within Excel and Word software (v.16, respectively; Microsoft. Redmond, WA, USA), solving discrepancies via a consensus. Datasets were gathered on the study’s first author, language and publication date, country, sample size, study design, type of oral surgery, CHX concentration and vehicle, control group’s intervention, recruitment and follow-up period, time intervals between checkup visits, sex, age, and outcomes of interest.

### 2.6. Evaluation of Quality and Risk of Bias

The authors critically appraised the methodological quality and risk of bias across primary-level studies using the updated Cochrane risk-of-bias tool (aka RoB2 tool) [30]. The following five potential bias domains were explored: (1) bias arising from the de-randomization process, (2) bias due to deviations from intended intervention, (3) bias due to missing outcome data, (4) bias in the measurement of the outcome, and (5) bias in the selection of reported result. After assessing these items, we identified each included study as low with some concerns or a high potential risk of bias for each domain.

Finally, an overall score was also estimated based on the following criteria: we rated the study as “low risk of bias” if the study was critically judged as having a potentially low risk of bias for all domains for this result; “some concerns” if the study was critically judged to raise some concerns in at least one domain for this result, but not at high risk of bias for any domain; and “high risk of bias” if the study was critically judged to be at a potential high risk of bias in at least one domain for this result or if the study was judged to have some concerns for multiple domains in a way that substantially lowered confidence in the result [30].

### 2.7. Statistical Analysis

Pain was analyzed using primary-level studies alongside a visual analog scale and reported as the absolute difference between the mean values of the study groups. As all results were expressed as continuous outcome measurements on the same scale, the means ± SD were extracted to calculate the mean difference (MD) with their corresponding 95% confidence intervals (CI). Data were expressed as medians, interquartile ranges, and/or maximum-minimum values, which were computed and transformed, if possible, into means ± SD using the methods proposed by Luo et al. (2018) and Wan et al. (2014) [31,32]. When data were only expressed graphically, extraction was performed using Engauge-Digitizer 4.1. If it was desirable to combine two or more different datasets expressed as the means ± SD from subgroups into a single group, the Cochrane Handbook formula was applied [27]. This meta-analysis was conducted using the inverse–variance method under a random-effects model (based on the DerSimonian and Laird method). This approach was *a priori* planned in our study protocol since considerable sources of clinical heterogeneity were expected (e.g., differences among surgical approaches, variations due to patients’ subjective perception of pain and the challenge of scoring it, etc.). The rest of the parameters expressed dichotomous outcomes, generally presenting low event rates and/or small sample sizes. Therefore, relative risks (RR) with 95% CIs were pooled using the Mantel–Haenszel method (fixed-effect model), which showed better statistical properties when data were sparse. These results were also re-expressed in terms of RR reduction (RRR = [1-RR] × 100%). Forest plots were constructed in all meta-analyses in order to graphically represent the effect sizes and for subsequent visual inspection analysis.

Heterogeneity between the studies was assessed using the χ^2^-based Cochran’s Q-test. Given the low statistical power of the Q-test, *p* < 0.10 was considered significant. We also applied the Higgins I^2^ statistic to estimate what proportion of variance in the observed effects reflected variation in true effects rather than a sampling error. The percentage of inter-study heterogeneity was quantified considering values of 50–75% as showcasing a moderate-to-high degree of inconsistency [33,34]. Preplanned subgroup meta-analyses were carried out to identify potential sources of heterogeneity and the influence of the specific study subpopulations (i.e., CHX association with other antiseptics or antibiotics, variations in vehicles or concentrations, type of oral surgery, and/or study design by parallel-group/split-mouth). Furthermore, small-study effect analyses were carried out to identify potential biases [35], such as a publication bias, the construction of funnel plots, and using the Egger regression test (performing a linear regression of the effect estimates on their standard errors, weighing using one/[variance of the effect estimate], considering a *p*_Egger_-value < 0.10 as significant) [36]. Stata software was used for all statistical analyses (v.16.1, Stata Corp, College Station, TX, USA).

## 3. Results

### 3.1. Results of the Literature Search

**Figure 1:**
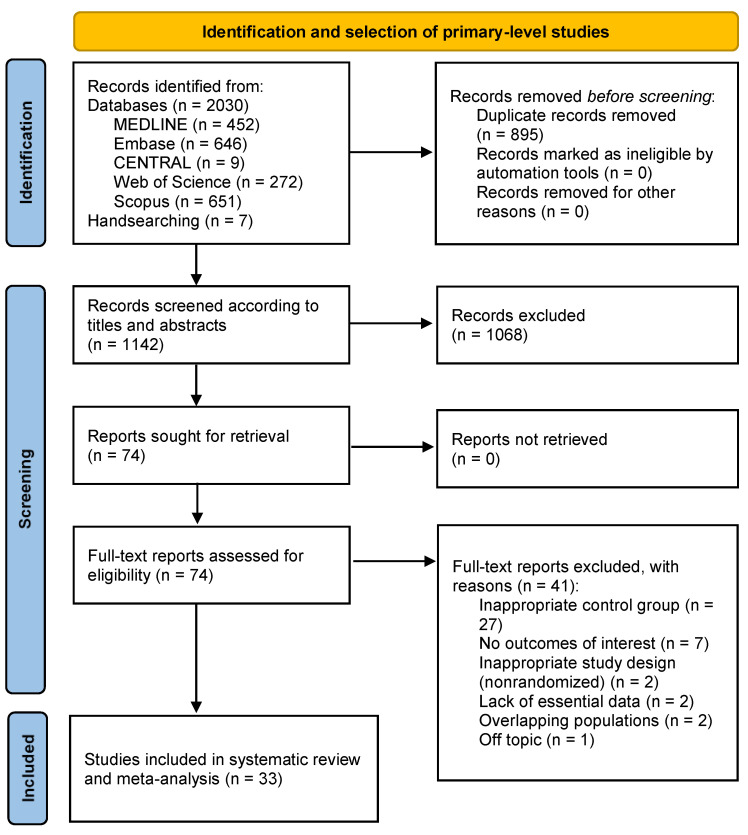
Flow diagram showing the identification and selection process of primary-level studies included in the present systematic review and meta-analysis.

The flow diagram (Figure 1) graphically depicts the searching and subsequent process of identification, screening, and selection studies. Overall, 2037 records were retrieved: 646 from Embase, 452 from MEDLINE, 651 from Scopus, 272 from Web of Science, 9 from CENTRAL, and 7 from hand-searching reference lists. After duplicates were removed, 1144 records were piloted and screened according to titles and abstracts, leaving a sample of 77 papers for full-text evaluation (in the Supplementary Information, exhibits the studies excluded jointly with their corresponding exclusion criteria). Finally, 33 studies meeting all eligibility criteria were included for qualitative evaluation and meta-analysis [37,38,39,40,41,42,43,44,45,46,47,48,49,50,51,52,53,54,55,56,57,58,59,60,61,62,63,64,65,66,67,68,69].

### 3.2. Study Characteristics

Table 1 summarizes the main characteristics of our study sample, and Table S2 (Supplementary Information) exhibits in detail the main variables gathered. These 33 primary-level studies analyzed a total of 4766 cases (2525 from the intervention group and 2241 controls), ranging between 20 and 744. Twenty-three studies were designed as RCTs and ten as q-RCTs. In relation to the interventions under investigation, the single application of CHX was the most frequently used (*n* = 31), followed by its association with chitosan (*n* = 4) and antibiotics (*n* = 4). Twenty-one of them were applied in rinse form, and the rest in a gel vehicle (*n* = 18), while 0.2% and 0.12% CHX were the most common concentrations (*n* = 26 and *n* = 11, respectively). Finally, the oral surgery procedures investigated were third molar (*n* = 28) and simple tooth extractions (*n* = 4), periodontal surgery (*n* = 4), and oral biopsies (*n* = 4).

### 3.3. Qualitative Evaluation

**Figure 2:**
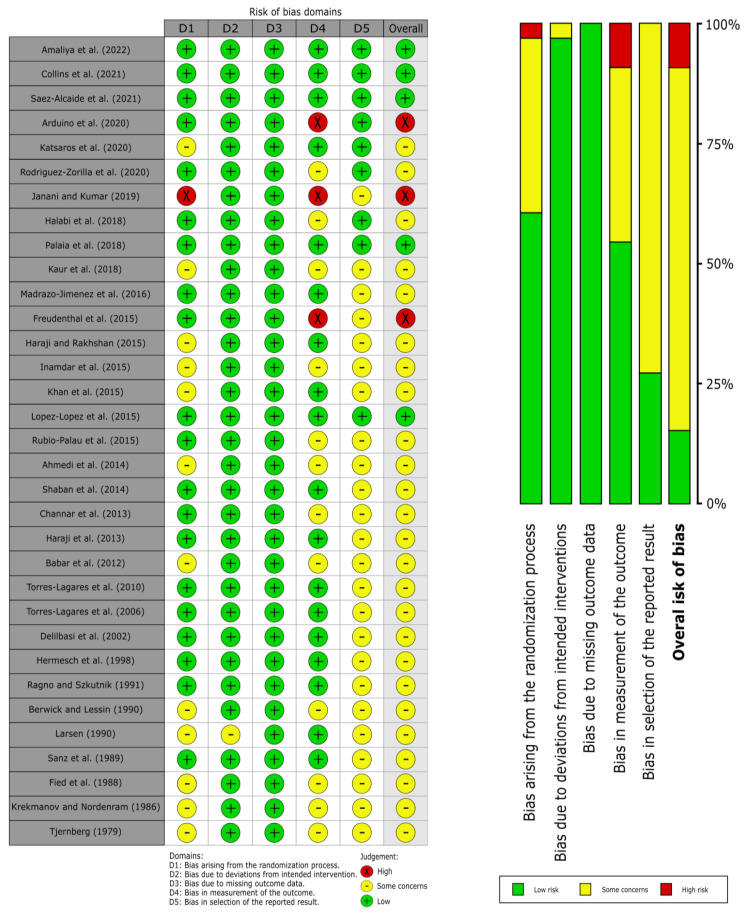
Quality plot graphically representing the risk of bias across primary-level studies and critically appraised using the RoB2 tool.

The evaluation of the risk of bias for each domain was carried out by applying the RoB2 tool (Figure 2) [30], and the following results were obtained:

Domain 1 (*bias arising from the randomization process*) obtained a low RoB in 60.61% of the studies, some concerns in 36.36%, and a high RoB in 3.03%. The most common bias was not specifying the randomization process of the studies since many of them only mentioned the study being randomized and did not provide more information on how said randomization was carried out. The most relevant bias was a failure to blind the allocation sequence (allocation sequence concealed).

Domain 2 (*bias due to deviations from intended intervention*) obtained a low RoB in 96.97% of the studies and some concerns in 3.03%. The most common bias did not provide clear information on whether participants and clinics knew the allocation of each group and whether that knowledge affected the results. The most relevant bias was the lack of information on whether any deviation from the planned study intervention occurred due to knowledge of the allocation sequence for each group and whether or not such deviation affected the results.

Domain 3 (*bias due to missing outcome data*) obtained a low RoB in 100% of the studies since all of them provided data on almost the entire studied cases; missing data had a size small enough to not affect the results.

Domain 4 *(bias in the measurement of the outcome*) obtained a low RoB in 54.55% of studies, some concerns in 36.36%, and a high RoB in 9.09%. The most relevant bias was using an inappropriate measurement method. The most frequent bias was that the person in charge of carrying out the measurements knew the assignment of each group, and the study did not provide clear information about the said knowledge.

Domain 5 (*bias in the selection of the reported result*) obtained a low RoB in 27.27% of the studies and some concerns in 72.73%. The most common and most relevant bias did not provide information on the existence of a prior analysis plan or protocol completed before the analysis of the results in order to compare whether the subsequent analysis was carried out according to that protocol or not.

### 3.4. Quantitative Evaluation

#### 3.4.1. Meta-Analysis on Wound Healing

**Figure 3:**
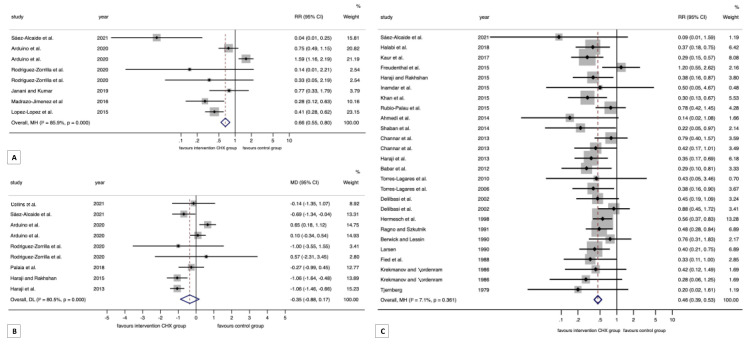
(**A**) Forest plot graphically represents the meta-analysis for the association between the application of CHX and wound healing. CHX, chlorhexidine; RR, relative risk; CI, confidence intervals. Fixed-effect model, Mantel–Haenszel method. An RR < 1 suggests that the application of CHX is associated with better wound healing. Diamonds indicate the pooled RRs with their corresponding 95% CIs. (**B**) The forest plot graphically represents a meta-analysis of the differences in pain between the CHX group and controls. CHX, chlorhexidine; MD, mean difference; CI, confidence intervals. Random-effects model, inverse-variance method. An MD < 0 suggests that pain levels were lower for the CHX group. Diamonds indicate the pooled MD with their corresponding 95% CIs. (**C**) The forest plot graphically represents the meta-analysis of the association between the application of CHX and alveolar osteitis. CHX, chlorhexidine; RR, relative risk; CI, confidence intervals. Fixed-effect model, Mantel–Haenszel method. An RR < 1 suggests that the application of CHX is associated with a lower risk of alveolar osteitis. Diamonds indicate the pooled RRs with their corresponding 95% CIs.

A significant association was found between the application of CHX and better wound healing (RR = 0.66, 95% CI = 0.55 to 0.80, *p* < 0.001; RRR = 34%), although a considerable degree of heterogeneity was observed (*p* < 0.001, I^2^ = 85.9%). More homogeneous subgroups were found after stratified meta-analyses and most of them preserved the statistically significant association (CHX + chitosan: RR = 0.25, 95% CI = 0.17 to 0.37, *p* < 0.001; CHX gel: RR = 0.26, 95% CI = 0.18 to 0.37, *p* < 0.001; CHX 0.12%: RR = 1.59, 95% CI = 1.16 to 2.19, *p* = 0.004; CHX 0.20%: RR = 0.41, 95% CI = 0.32 to 0.53, *p* < 0.001; third molar surgery: RR = 0.29, 95% CI = 0.21 to 0.41, *p* < 0.001; parallel-group design: RR = 0.70, 95% CI = 0.57 to 0.86, *p* = 0.001; split-mouth design: RR = 0.41, 95% CI = 0.23 to 0.74, *p* = 0.003; low RoB: RR = 0.26, 95% CI = 0.17 to 0.40, *p* < 0.001; Some concerns RoB: RR = 0.24, 95% CI = 0.11 to 0.53, *p* < 0.001) (Table 2, Figure 3A and Figures S1–S6).

#### 3.4.2. Meta-Analysis on Alveolar Osteitis

A significant association after the application of CHX and a lower risk of alveolar osteitis (RR = 0.46, 95% CI = 0.39 to 0.53, *p* < 0.001) considerably reduced the incidence of alveolar osteitis when compared with the controls (RRR = 54%) and obtained homogeneous results across primary-level studies (heterogeneity: *p* = 0.36, I^2^ = 7.1%). Several subgroups also maintained this statically significant result (CHX single: RR = 0.49, 95% CI = 0.41 to 0.57, *p* < 0.001; CHX + antibiotics: RR = 0.35, 95% CI = 0.23 to 0.54, *p* < 0.001; CHX + chitosan: RR = 0.09, 95% CI = 0.01 to 1.59, *p* < 0.001; CHX gel: RR = 0.40, 95% CI = 0.31 to 0.51, *p* < 0.001; CHX rinse: RR = 0.50, 95% CI = 0.41 to 0.62, *p* < 0.001; CHX 0.12%: RR = 0.47, 95% CI = 0.37 to 0.59, *p* < 0.001; CHX 0.20%: RR = 0.46, 95% CI = 0.37 to 0.57, *p* < 0.001; third molar: RR = 0.48, 95% CI = 0.40 to 0.56, *p* < 0.001; simple extraction: RR = 0.34, 95% CI = 0.21 to 0.54, *p* < 0.001; parallel-group design: RR = 0.50, 95% CI = 0.42 to 0.60, *p* < 0.001; split-mouth design: RR = 0.35, 95% CI = 0.25 to 0.48, *p* < 0.001; Some concerns RoB: RR = 0.47, 95% CI = 0.38 to 0.52, *p* < 0.001) (Table 2, Figure 3C and Figures S7–S12).

#### 3.4.3. Meta-Analysis on Erythema

A significant association was found between the application of CHX and better healing, with fewer erythematous wounds (RR = 0.60, 95% CI = 0.39 to 0.93, *p* = 0.02; RRR = 40%), although a considerable degree of heterogeneity was observed (*p* = 0.02, I^2^ = 76.3%) (Table 2, Figure S13). Subgroup meta-analyses were not performed for this variable, where only three primary-level studies entered into the meta-analysis.

#### 3.4.4. Meta-Analysis on Epithelization

A significant association was not found between the application of CHX and epithelization (RR = 1.05, 95% CI = 0.77 to 1.42, *p* = 0.76), and moderate heterogeneity was also observed (*p* = 0.06, I^2^ = 55.6%) (Table 2, Figure S14). Subgroup meta-analyses were not performed for this parameter, where only five primary-level studies entered into the meta-analysis.

#### 3.4.5. Meta-Analysis on Pain during Wound Healing

Pain levels were not significantly different in the CHX group compared to the controls using a visual analog scale (MD = −0.35, 95% CI = −0.88 to 0.17, *p* = 0.19), which also showed significant heterogeneity (*p* < 0.001, I^2^ = 80.5%) (Table 2, Figure 3B and Figures S15–S20). More homogeneous subgroups were found after the stratified meta-analyses, and some of them showed significant differences, indicating slightly less pain in the CHX group (CHX gel: MD = −0.97, 95% CI = −1.26 to −0.68, *p* < 0.001; Split-mouth design: MD = −1.06, 95% CI = −1.39 to −0.73, *p* < 0.001; Low RoB: MD = −0.45, 95% CI = −0.90 to −0.003, *p* = 0.05; Some concerns RoB: MD = −1.04, 95% CI = −1.36 to −0.71, *p* < 0.001).

#### 3.4.6. Small-Study Effects Analysis

The visual examination of funnel plots’ asymmetry and the corresponding statistical tests were run with the same purpose affirmed for the absence of small-study effects for the variables wound healing (*p*_Egger_ = 0.11) and pain (*p*_Egger_ = 0.96), except for alveolar osteitis (*p*_Egger_ = 0.03) for which biases, e.g., publication bias, could not be ruled out (Figures S21–S23).

**Table 2:** Meta-analysis of the efficacy of chlorhexidine on wound healing after oral surgery procedures.

					Pooled Data		Heterogeneity	
Meta-Analyses	No. of Studies *	No. of Cases *	Stat. Model	Wt	ES (95% CI)	*p*-Value	*p*_het_	I^2^ (%)
**Wound healing**								
all (poor vs. better wound healing) ^a^	8	771	FEM	M-H	RR = 0.66 (0.55 to 0.80)	<0.001	<0.001	85.9
Subgroup analysis by type of intervention ^b^						<0.001 ^c^		
CHX single	4	527	FEM	M-H	RR = 1.10 (0.87 to 1.40)	0.43	0.02	71.3
CHX + chitosan	4	244	FEM	M-H	RR = 0.25 (0.17 to 0.37)	<0.001	0.02	70.8
Subgroup analysis by type of vehicle ^b^						<0.001 ^c^		
CHX gel	5	272	FEM	M-H	RR = 0.26 (0.18 to 0.37)	<0.001	0.04	60.1
CHX rinse	3	499	FEM	M-H	RR = 1.14 (0.90 to 1.45)	0.28	0.01	77
Subgroup analysis by type of concentration ^b^						<0.001 ^c^		
CHX 0.12%	1	239	—	—	RR = 1.59 (1.16 to 2.19)	0.004	—	—
CHX 0.20%	7	532	FEM	M-H	RR = 0.41 (0.32 to 0.53)	<0.001	0.007	66.0
Subgroup analysis by type of oral surgery						<0.001 ^c^		
biopsy	2	474	FEM	M-H	RR = 1.18 (0.92 to 1.51)	0.20	0.005	87.0
third molar	6	297	FEM	M-H	RR = 0.29 (0.21 to 0.41)	<0.001	0.02	62.2
Subgroup analysis by study design						0.09 ^c^		
Parallel-group design	6	696	FEM	M-H	RR = 0.70 (0.57 to 0.86)	0.001	<0.001	88.4
Split-mouth design	2	75	FEM	M-H	RR = 0.41 (0.23 to 0.74)	0.003	0.08	67.3
Subgroup analysis by overall RoB						<0.001 ^c^		
Low	2	166	FEM	M-H	RR = 0.26 (0.17 to 0.40)	<0.001	0.002	89.3
Some concerns	3	106	FEM	M-H	RR = 0.24 (0.11 to 0.53)	<0.001	0.83	0.0
High	3	166	FEM	M-H	RR = 1.14 (0.90 to 1.45)	0.28	0.01	77.0
**Alveolar osteitis**								
All (alveolar osteitis vs. healing) ^a^	26	4205	FEM	M-H	RR = 0.46 (0.39 to 0.53)	<0.001	0.36	7.1
Subgroup analysis by type of intervention ^b^						0.17 ^c^		
CHX single	21	3504	FEM	M-H	RR = 0.49 (0.41 to 0.57)	<0.001	0.32	10.7
CHX + antibiotics	4	629	FEM	M-H	RR = 0.35 (0.23 to 0.54)	<0.001	0.84	0.0
CHX + chitosan	1	72	—	—	RR = 0.09 (0.01 to 1.59)	<0.001	—	—
Subgroup analysis by type of vehicle ^b^						<0.14 ^c^		
CHX gel	13	1523	FEM	M-H	RR = 0.40 (0.31 to 0.51)	<0.001	0.17	27.5
CHX rinse	13	2682	FEM	M-H	RR = 0.50 (0.41 to 0.62)	<0.001	0.71	0.0
Subgroup analysis by type of concentration ^b^						0.52 ^c^		
CHX 0.12%	7	1936	FEM	M-H	RR = 0.47 (0.37 to 0.59)	<0.001	0.78	0.0
CHX 0.20%	18	2219	FEM	M-H	RR = 0.46 (0.37 to 0.57)	<0.001	0.18	23.6
CHX 1%	1	50	—	—	RR = 0.14 (0.02 to 1.08)	0.06	—	—
Subgroup analysis by type of oral surgery ^b^						0.17 ^c^		
Third molar	23	2992	FEM	M-H	RR = 0.48 (0.40 to 0.56)	<0.001	0.33	9.4
Simple extraction	3	1213	FEM	M-H	RR = 0.34 (0.21 to 0.54)	<0.001	0.92	0.0
Subgroup analysis by study design						0.051 ^c^		
Parallel-group design	20	3363	FEM	M-H	RR = 0.50 (0.42 to 0.60)	<0.001	0.39	5.4
Split-mouth design	6	842	FEM	M-H	RR = 0.35 (0.25 to 0.48)	<0.001	0.75	0.0
Subgroup analysis by overall RoB ^b^						—		
Low	1	72	—	—	RR = 0.09 (0.01 to 1.59)	0.99	—	—
Some concerns	24	4038	FEM	M-H	RR = 0.47 (0.38 to 0.52)	<0.001	0.64	0.0
High	1	95	—	—	RR = 1.20 (0.55 to 2.62)	0.65	—	—
**Erythema**								
All (erythematous vs. better healing) ^a^	3	88	FEM	M-H	RR = 0.60 (0.39 to 0.93)	0.02	0.02	76.3
**Epithelization**								
All (not-epithelized vs. epithelized) ^a^	5	140	FEM	M-H	RR = 1.05 (0.77 to 1.42)	0.76	0.06	55.6
**Dehiscence**								
All (open vs. closed)	1	25	—	—	RR = 0.77 (0.33 to 1.79)	0.55	—	—
**Pain**								
All (absolute difference) ^a^	9	919	REM	D-L	MD = −0.35 (−0.88 to 0.17)	0.19	<0.001	80.5
Subgroup analysis by type of intervention ^b^						0.52 ^c^		
CHX single	7	819	REM	D-L	MD = −0.33 (−0.95 to 0.28)	0.29	<0.001	84.9
CHX + chitosan	2	100	REM	D-L	MD = −0.63 (−1.26 to 0.01)	0.052	0.40	0.0
Subgroup analysis by type of vehicle ^b^						<0.001 ^c^		
CHX gel	5	378	REM	D-L	MD = −0.97 (−1.26 to−0.68)	<0.001	0.72	0.0
CHX rinse	4	541	REM	D-L	MD = 0.18 (−0.25 to 0.60)	0.41	0.13	46.8
Subgroup analysis by type of concentration ^b^						0.71 ^c^		
CHX 0.12%	3	436	REM	D-L	MD = −0.19 (−1.48 to 1.10)	0.77	<0.001	93.3
CHX 0.20%	6	483	REM	D-L	MD = −0.45 (−0.95 to 0.05)	0.08	0.05	56.0
Subgroup analysis by type of oral surgery ^b^						<0.001 ^c^		
Biopsy	3	504	REM	D-L	MD = 0.21 (−0.29 to 0.70)	0.41	0.07	61.9
Periodontal	1	37	—	—	MD = −0.14 (−1.35 to 1.07)	0.82	—	—
Third molar	5	378	REM	D-L	MD = −0.97 (−1.26 to −0.68)	<0.001	0.72	0.0
Subgroup analysis by study design ^b^						<0.001 ^c^		
Parallel-group design	7	669	REM	D-L	MD = −0.03 (−0.48 to 0.42)	0.90	0.05	53.4
Split-mouth design	2	250	REM	D-L	MD = −1.06 (−1.39 to −0.73)	<0.001	0.99	0.0
Subgroup analysis by overall RoB ^b^						<0.001 ^c^		
Low	3	138	REM	D-L	MD = −0.45 (−0.90 to −0.003)	0.05	0.60	0.0
Some concerns	4	306	REM	D-L	MD = −1.04 (−1.36 to −0.71)	<0.001	0.75	0.0
High	2	475	REM	D-L	MD = 0.37 (−0.17 to 0.91)	0.18	0.10	64.1

Abbreviations: Stat., statistical; Wt, method of weighting; M-H, Mantel–Haenszel method; D-L, DerSimonian and Laird method; ES, effect size; FEM, fixed-effect model; REM, random-effects model; RR, relative risk; MD, mean difference; CI, confidence intervals; RoB, risk of bias. *—Note that more than one analysis unit was analyzed per study. a—Meta-analysis. b—Subgroup meta-analysis. c—Test for between-subgroup differences.

## 4. Discussion

The present systematic review and meta-analysis carried out on 33 studies and 4766 cases provides evidence-based results on the benefits of using CHX after oral surgical procedures. The application of CHX was 1.52 times more effective (RR = 0.66, 95% CI = 0.55 to 0.80, *p* < 0.001) in the healing of oral surgical wounds compared to the controls in which CHX was not used; this translates into 34% of patients who underwent oral surgery treated with CHX presenting with a significant reduction in the risk of developing surgical complications and/or having poor surgical wound healing. The benefits of applying CHX probably derive from its antimicrobial effect mediated by its control of biofilm through a reduction in the oral pathogen load [61]. The development of infections has been directly associated with delayed wound healing, which is due to local inflammation, the inhibition of angiogenesis, decreased epithelization, and delayed final tissue remodeling [12]. Therefore, the use of antiseptic agents is necessary for better microbial control of wounds after oral surgery. In this sense, our meta-analysis confirmed that CHX is an effective agent for this purpose, with broad additional benefits for clinical practice derived from its low cost, easy application, and safety, which makes its routine and use possible; an additional advantage is its availability in different vehicles [23]. In relation to the forms of application, our study has also demonstrated a significantly higher efficacy for the use of CHX in gel vs. the use of CHX in rinses (*p* < 0.001), at a concentration of 0.20% vs. 0.12% (*p* < 0.001). The use of CHX gel appears to exert a more extended pharmacological activity than rinse at the point of application [70], despite the fact that in this form of application, the contact time of the drug with the lesion cannot be effectively controlled; however, in cases of deep wounds that are difficult to access, the patient may have some difficulty in applying the drug adequately. On the other hand, the greater efficacy of CHX at a concentration of 0.20% is justified by its demonstrated dose-dependent effect, with bacteriostatic properties at low doses and bactericidal properties at high concentrations [24,25].

Interestingly, in our meta-analysis, oral wound healing was much higher in the subgroup of patients in which CHX was associated with chitosan, reaching a benefit of wound healing that was four times higher than that observed in the controls that did not use CHX plus chitosan; this translates into a reduced risk of complications and/or poor healing in 75% of the cases. In spite of the fact that there are not a large number of clinical trials that justify this result (*n* = 4), a large magnitude of the effect observed (RR = 0.25) and the narrow confidence interval obtained in the analysis (95% CI = 0.17 to 0.37) certify the robustness of this result. Furthermore, the use of CHX plus chitosan showed significantly higher efficacy than the use of CHX alone (*p* < 0.001); all this provides high-quality scientific evidence indicating that the joint use of CHX and chitosan should be recommended in clinical practice. The efficacy of chitosan has been contrasted in vitro in preclinical studies [71,72], in vivo by animal experimentation [73], and in clinical research with patients, where beneficial properties such as improved epithelialization have also been observed [74,75,76] alongside the activation of neoangiogenesis, promotion of fibroblastic colonization [77,78], and acceleration of wound healing time [79,80].

Another important result of our meta-analysis demonstrates the efficacy of CHX in preventing the development of alveolar osteitis. In cases in which CHX was applied, both in gel and rinses, the incidence of alveolar osteitis after any type of exodontia was 2.17 times lower than in the controls, implying that 54% of patients presented a decreased risk of developing this complication; the magnitude of this effect (RR = 0.46) together with the narrow confidence interval obtained (95% CI = 0.39–0.53) points to the robustness of this statistically significant result (*p* < 0.001). This result has been confirmed in previous meta-analytical studies [81,82,83,84,85], although our meta-analysis, by including twice as many studies and cases as those collected by any of the preceding meta-analyses, reinforces evidence of the reported results and advises the routine use of CHX after tooth extractions, since it is known that the development of alveolar osteitis is a serious complication associated with severe pain and impaired quality of life for patients [86,87]. Only one study addresses the effect of the combined use of CHX (0.20%) and chitosan applied as a gel in the prevention of alveolar osteitis after surgical third-molar extractions [41]. Although a single clinical trial cannot support solid evidence, the results show a remarkable trend effect that makes it advisable to further investigate this research line.

Our meta-analysis also indicates that the application of CHX in gel form (no data on rinses) generated a significant decrease in post-surgical pain after third molar surgery compared to the controls (MD = −0.97, 95% CI = −1.26 to −0.68, *p* < 0.001). Moreover, the statistical results were homogeneous (*p*_het_ = 0.72, I^2^ = 0.0%), confirming that this effect is consistent and robust. This decrease in pain was also significantly higher after the application of CHX with third molar surgery compared to periodontal surgery and biopsy (*p* < 0.001). Again, in this aspect, evidence on the goodness of using CHX plus chitosan for the prevention of pain after third molar surgery derives exclusively from two clinical trials which, although by definition do not support solid evidence, yield a result very close to significance (*p* = 0.052), suggesting that new research studies probably allow confirmation of the benefits regarding the combination of both drugs.

Although all primary-level studies included in the present systematic review had the same study design (i.e., randomized clinical trials), not all of them were designed with the same conscientiousness. The domain that had the higher risk of bias was bias in the selection of the reported result (72.73% as some concerns) since many studies did not mention having a previous protocol with which to compare the methodology used to see if the measurements were made from different methods; instead, the most favorable ones were chosen for the study results. After stratifying the meta-analysis by overall study quality, we could also demonstrate that the subgroup of studies that had a better methodological design and quality identified significant differences for the parameters of wound healing, alveolar osteitis, and pain, as well as a larger effect size. This led us to confirm that the better the study was designed, the better it could demonstrate the efficacy of CHX in wound healing. Therefore, clinical trials focusing on this research topic should be more rigorous in the methodological design in order to reduce the risk of bias, obtain reliable results, and standardize future research. Future studies should preferably be prospective RCTs with a pre-established full trial protocol—accessible, precise, and transparent—and a larger sample size. Furthermore, they should standardize the clinical procedures for the application of CHX, the postoperative follow-up periods, as well as the criteria used to determine the efficacy of this drug on oral wound healing.

Some potential limitations of our systematic reviews and meta-analysis should also be discussed. First, considerable clinical heterogeneity sources were expected, and some degree of statistical heterogeneity was also demonstrated through meta-analytical techniques for the parameters of wound healing and pain. In order to overcome this limitation, stratified meta-analyses were conducted, showing more homogeneous subgroups of studies and confirming the potential sources of heterogeneity (e.g., the relevance of the CHX vehicle -gel vs. rinse- or variations due to different surgical approaches). Second, the presence of publication bias could not be ruled out for all parameters. Unfortunately, the trend of mostly positive results when published—rejecting findings on the basis of the direction or strength of the study results—constitutes a challenge that is hard to overcome in the current biomedical sciences era. Finally, an inherent limitation of primary-level studies included in this systematic review—highlighted after our risk of bias critical appraisal and data analysis—was the failure to report important information and datasets (e.g., within-patient correlations in split-mouth study designs). This drawback limited the number of observations gathered and challenged the performance of relevant adjusted secondary analyses through subgroup meta-analyses or meta-regressions. Given the clinical and methodological importance of these reflections, we encourage future studies to communicate their datasets in a more rigorous way, preferably reporting individual patient data. Despite the above limitations, the robust nature of the present study is remarkable, providing relevant findings with direct translational applicability to clinical practice, analyzing the effect of CHX on oral wound healing in a comprehensive approach on a large sample of primary-level studies and patients using meta-analytical techniques.

## 5. Conclusions

In conclusion, the results of our systematic review and meta-analysis demonstrate evidence that the application of CHX exerts a beneficial effect on oral surgical wound healing, significantly decreasing patients’ risk of developing surgical complications and/or poor wound healing. This benefit is greater when CHX is used at 0.20% in gel form. Furthermore, it has been shown that the addition of chitosan to CHX significantly increases its beneficial effect on surgical wound healing; therefore, the use of CHX plus chitosan for the preventive treatment of oral surgical wounds should be advised. The use of CHX has been especially beneficial in the prevention of alveolar osteitis after any type of dental extraction, and it has also been effective—when applied as a gel—in the reduction in pain after the surgical extraction of third molars. There is insufficient evidence, due to scarce research on the efficacy of the joint use of CHX and chitosan for the prevention of alveolar osteitis and pain after dental extractions, although the results of a few clinical trials on the subject seem to indicate a favorable effect, and it is advisable to increase research in this regard.

## Figures and Tables

**Table 1:** Summarized study characteristics.

Total	33 Studies
Year of publication	1979–2022
Number of cases	
Total	4766 cases
Intervention group	2525 cases
Control group	2241 cases
Sample size, range	20 to 744 cases
Type of interventions *	
CHX	31 studies
CHX + chitosan	4 studies
CHX + antibiotics	4 studies
Type of CHX vehicles *	
Gel	18 studies
Rinse	21 studies
Type of CHX concentrations *	
1%	1 study
0.20%	26 studies
0.12%	11 studies
0.006%	1 study
Type of oral surgery procedures *	
Third molar surgery	28 studies
Simple tooth extraction	4 studies
Periodontal surgery	4 studies
Oral biopsy	3 studies
Type of study design *	
Parallel group design	31 studies
Split-mouth design	8 studies

* Note that six primary-level studies reported two analysis units (total *n* = 39).

## Data Availability

Data are contained within the article or Supplementary Material.

## Supplementary Materials

The following supporting information can be downloaded at: https://www.mdpi.com/article/10.3390/antibiotics12101552/s1. Table S1. Search strategy for each database, number of results, and execution date; Table S2. Characteristics of analyzed studies (*n* = 33); Figure S1. Forest plot graphically representing the stratified meta-analysis by type of intervention on the association between the application of CHX and wound healing; Figure S2. Forest plot graphically representing the stratified meta-analysis by type of vehicle on the association between the application of CHX and wound healing; Figure S3. Forest plot graphically representing the stratified meta-analysis by type of concentration on the association between the application of CHX and wound healing; Figure S4. Forest plot graphically representing the stratified meta-analysis by type of oral surgery on the association between the application of CHX and wound healing; Figure S5. Forest plot graphically representing the stratified meta-analysis by study design on the association between the application of CHX and wound healing; Figure S6. Forest plot graphically representing the stratified meta-analysis by overall RoB on the association between the application of CHX and wound healing; Figure S7. Forest plot graphically representing the stratified meta-analysis by type of intervention on the association between the application of CHX and alveolar osteitis; Figure S8. Forest plot graphically representing the stratified meta-analysis by type of vehicle on the association between the application of CHX and alveolar osteitis; Figure S9. Forest plot graphically representing the stratified meta-analysis by type of concentration on the association between the application of CHX and alveolar osteitis; Figure S10. Forest plot graphically representing the stratified meta-analysis by type of oral surgery on the association between the application of CHX and alveolar osteitis; Figure S11. Forest plot graphically representing the stratified meta-analysis by type of study design on the association between the application of CHX and alveolar osteitis; Figure S12. Forest plot graphically representing the stratified meta-analysis by overall RoB on the association between the application of CHX and alveolar osteitis; Figure S13. Forest plot graphically representing the meta-analysis on the association between the application of CHX and wound erythema; Figure S14. Forest plot graphically representing the meta-analysis on the association between the application of CHX and wound epithelization; Figure S15. Forest plot graphically representing the stratified meta-analysis by type of intervention on the differences in pain between the CHX group and controls; Figure S16. Forest plot graphically representing the stratified meta-analysis by type of vehicle on the differences in pain between the CHX group and controls; Figure S17. Forest plot graphically representing the stratified meta-analysis by type of concentration on the differences in pain between the CHX group and controls; Figure S18. Forest plot graphically representing the stratified meta-analysis by type of oral surgery on the differences in pain between the CHX group and controls; Figure S19. Forest plot graphically representing the stratified meta-analysis by type of study design on the differences in pain between the CHX group and controls; Figure S20. Forest plot graphically representing the stratified meta-analysis by overall RoB on the differences in pain between the CHX group and controls; Figure S21. A funnel plot of estimated effect sizes against their standard errors, graphically representing the analysis of small-study effects on the association between the application of CHX and wound healing; Figure S22. A funnel plot of estimated effect sizes against their standard errors, graphically representing the analysis of small-study effects on the association between the application of CHX and alveolar osteitis; Figure S23. A funnel plot of the differences in pain between CHX group and controls, expressed as mean differences against their standard errors; List of full-text articles excluded with reasons.
