# Supplementary material for: Efficacy of Chlorhexidine after Oral Surgery Procedures on Wound Healing: Systematic Review and Meta-Analysis
Source: Antibiotics (Basel). 2023 Oct 20;12(10):1552. doi: 10.3390/antibiotics12101552 (PMC10604691; doi:10.3390/antibiotics12101552)
Supplement: Supplementary file 1 [file antibiotics-12-01552-s001.zip › antibiotics-2651130-supplementary.pdf]

Supplementary information to the manuscript

Efficacy of Chlorhexidine after oral surgery procedures on wound healing: systematic review and meta-analysis

María de Nuria Romero-Olid[1,2], Elena Bucataru[1,2], Pablo Ramos-García[1,2,*] and Miguel Ángel González-Moles[1,2]

1- School of Dentistry, University of Granada, 18071 Granada, Spain

2- Instituto de Investigación Biosanitaria ibs.GRANADA, 18012 Granada, Spain

*__Corresponding Author:__

Pablo Ramos-García (pabloramos@ugr.es)

Table of contents

1. Search strategy. Table S1. 3
2. Characteristics of analyzed studies. Table S2 4
3. Meta-analysis on wound healing 5
 3.1 Subgroup meta-analysis by type of intervention..........5
 3.2 Subgroup meta-analysis by type of vehicle 6
 3.3 Subgroup meta-analysis by type of concentration 7
 3.4 Subgroup meta-analysis by type of oral surgery 8
 3.5 Subgroup meta-analysis by overall RoB..........9
4. Meta-analysis on alveolar osteitis. 10
 4.1 Subgroup meta-analysis by type of intervention..........10
 4.2 Subgroup meta-analysis by type of vehicle 11
 4.3 Subgroup meta-analysis by type of concentration 12
 4.4 Subgroup meta-analysis by type of oral surgery 13
 4.5 Subgroup meta-analysis by overall RoB..........14
5. Meta-analysis on wound erythema 15
6. Meta-analysis on wound epithelization 16
....
7. Meta-analysis on pain...........17
 7.1 Subgroup meta-analysis by type of intervention..........17
 7.2 Subgroup meta-analysis by type of vehicle 18
 7.3 Subgroup meta-analysis by type of concentration 19
 7.4 Subgroup meta-analysis by type of oral surgery 20
 7.5 Subgroup meta-analysis by overall RoB..........21
8. Analysis of small-study effects..........22
 8.1 Wound healing 22
 8.2 Alveolar osteitis 23
 8.3 Pain..........24
9. List of excluded studies with reasons 25

1. Search strategy

Table S1. Search strategy for each database, number of results, and execution date.

	Query/Search Strategy	Results/ Items found	Search time limits
MEDLINE PubMed	("Wound Healing"[Mesh] OR "wound"[All Fields] OR "healing"[All Fields] OR "cicatrization"[All fields] OR "re-epithelialization"[All fields] OR "mucosal recovery"[All Fields] OR "Dry Socket"[Mesh] OR “alveolar osteitis”[All Fields] OR “dry socket”[All Fields] OR “alveolitis sicca dolorosa”[All Fields] OR “fibrinolytic alveolitis”[All Fields]) AND ("mouth"[MeSH] OR "mouth"[All Fields] OR "oral"[All Fields]) AND ("Chlorhexidine"[Mesh] OR "chlorhexidine"[All fields])	452	January, 2023
Embase	('wound healing'/exp OR 'wound' OR 'healing' OR 'cicatrization' OR 're-Epithelialization' OR 'mucosal recovery' OR 'alveolar osteitis' OR 'dry socket' OR 'alveolitis sicca dolorosa' OR 'fibrinolytic alveolitis') AND ('mouth'/exp OR 'mouth') AND ('chlorhexidine'/exp OR 'chlorhexidine')	646	January, 2023
Web of Science	TS=("wound healing" OR "wound" OR "healing" OR "cicatrization" OR "re-Epithelialization" OR "mucosal recovery" OR "alveolar osteitis" OR "dry socket" OR "alveolitis sicca dolorosa" OR "fibrinolytic alveolitis") AND TS=("mouth" OR "oral") AND TS=("chlorhexidine")	272	January, 2023
Scopus	TITLE-ABS-KEY(("wound healing" OR "wound" OR "healing" OR "cicatrization" OR "re-Epithelialization" OR "mucosal recovery" OR "alveolar osteitis" OR "dry socket" OR "alveolitis sicca dolorosa" OR "fibrinolytic alveolitis") AND ("mouth" OR "oral") AND "chlorhexidine")	651	January, 2023
CENTRAL	#1 MeSH descriptor: [Wound Healing] explode all trees<br>#2 MeSH descriptor: [Mouth] explode all trees<br>#3 MeSH descriptor: [Chlorhexidine] explode all trees<br>#4 #1 AND #2 AND #3	9	January, 2023
Total	2030		

2. Table S2. Characteristics of analyzed studies (n = 33).

Study (year)	Country	Study design	Sample, n (I/C)	Sex m/f	Age, y mean±SD (range)	Intervention group (CHX form)	Control group	Oral Surgery	Study parameters
Amaliya *et al.* (2022)	Indonesia	Parallel-group RCT	32 (16/16)	11/21	26.09±8.51 (19-46)	0.20% CHX (gel) + 500 mg of mefenamic acid	Placebo gel + 500 mg of mefenamic acid	Unilateral extraction of 1M	-Erythema -Epithelization
Collins *et al.* (2021)	Dominican Republic	Parallel-group RCT	37 (17/20)	18/19	48.95±11.38 (30-68)	0.12% CHX (rinse)	NaCl	Periodontal Surgery	-Pain
Sáez-Alcaide *et al.* (2021)	Spain	Parallel-group RCT	72 (36/36)	13/23	22.94±2.67 (nr)	0.20% chlorhexidine, chitosan (gel)	Placebo gel	Lower 3M surgery	-Alveolar osteitis -Wound healing -Pain
Arduino *et al.* (2020)	Italy	Parallel-group RCT	354 (118/115/121)	133/221	57.40±16.3 (nr)	0.12% CHX (rinse) 0.20% CHX (rinse)	No intervention	Oral mucosal biopsy	-Wound healing -Pain
Katsaros *et al.* (2020)	USA	Parallel-group q-RCT	35 (11/13/11)	22/26 missing = 13	49.50±nr (18-65)	0.12% CHX (rinse) 5% dilution of 0.12% CHX (rinse)	Water	Periodontal surgery	-Epithelization
Rodriguez-Zorrilla *et al.* (2020)	Spain	Parallel-group RCT	35 (7/7/21)	5/16	24.50±4.10 (18-37)	0.20% CHX (gel) 0.20% CHX + chitosan (gel)	Placebo	Surgical extraction of inferior 3M (bilateral)	-Wound healing -Erythema -Epithelization -Pain
Janani and Kumar (2019)	India	Split-mouth q-RCT	25 (12/13)	14/11	nr (18-45)	0.20% CHX (rinse)	NaCl	Surgical extraction of 3M	-Wound healing -Dehiscence
Halabi *et al.* (2018)	Chile	Parallel-group RCT	744 (372/372)	363/381	43.43±14.99 (nr)	0.12% CHX (rinse)	Placebo	Simple tooth extraction	-Alveolar osteitis
Palaia *et al.* (2018)	Italy	Parallel-group RCT	29 (15/14)	19/37	56.8±15.49 54.2±17.29 (14-81)	0.20% CHX (rinse)	Placebo	Oral mucosal biopsy	-Pain
Kaur *et al.* (2017)	India	Split-mouth q-RCT	300 (150/150)	86/64	30.50±2.50 (20-45)	0.20% CHX (gel) + antibiotic	Placebo	Bilateral extraction of 3M	-Alveolar osteitis
Madrazo-Jimenez *et al.* (2016)	Spain	Split-mouth RCT	50 (25/25)	15/10	26.47±6.74 (18-45)	0.20% chlorhexidine, chitosan (gel)	No intervention	3M	-Wound healing
Freudenthal *et al.* (2015)	Sweden	Parallel-group RCT	95 (48/47)	45/50	33±10.30 (19-65)	0.20% CHX (gel)	Placebo	3M	-Alveolar osteitis
Haraji and Rakhshan (2015)	Iran	Split-mouth q-RCT	90 (45/45)	24/21	22.09±2.79 (17-31)	0.20% CHX (gel on gelatin sponge)	Dry gelatin sponge	3M	-Alveolar osteitis -Pain
Inamdar *et al.* (2015)	Saudi Arabia	Parallel-group q-RCT	20 (10/10)	17/13	28.15±6.21 (18-60)	0.20% CHX (gel)	No intervention	3M	-Alveolar osteitis
Khan *et al.* (2015)	Pakistan	Parallel-group RCT	253 (128/125)	106/147	36.65±11 (nr)	0.20% CHX (gel)	Placebo	Simple tooth extraction	-Alveolar osteitis
Lopez-Lopez *et al.* (2015)	Spain	Parallel-group RCT	94 (47/47)	22/25	34±7 (19-47)	0.20% chlorhexidine, chitosan (gel)	Bicarbonate	3M	-Wound healing
Rubio-Palau *et al.* (2015)	Spain	Parallel-group RCT	160 (80/80)	74/86	25.04±nr (nr)	0.20% CHX (gel)	Placebo	3M	-Alveolar osteitis

Ahmedi *et al.* (2014)	Republic of Kosovo	Split-mouth q-RCT	50 (25/25)	nr	nr (18-30)	1% CHX (gel)	NaCl	3M	-Alveolar osteitis
Shaban *et al.* (2014)	Iran	Split-mouth RCT	82 (41/41)	14/27	24.15±5.02 (nr)	0.20% CHX (gel)	No intervention	3M	-Alveolar osteitis
Channar *et al.* (2013)	India	Parallel-group RCT	214 (73/69/72)	129/85	30.44±5.20 (24-40)	0.20% CHX (rinse)<br>0.20% CHX (rinse) + antibiotics	NaCl	3M	-Alveolar osteitis
Haraji *et al.* (2013)	Iran	Split-mouth RCT	160 (80/80)	39/41	21.60±2.50 (17-31)	0.12% CHX (gel)	Dry sponge	3M	-Alveolar osteitis<br>-Pain
Babar *et al.* (2012)	Pakistan	Parallel-group q-RCT	100 (50/50)	65/35	29±6 (18-40)	0.20% CHX (gel)	No intervention	3M	-Alveolar osteitis
Torres-Lagares *et al.* (2010)	Spain	Parallel-group RCT	38 (14/24)	33/5	32.18±13.63 (18-57)	0.12% CHX (gel)	Placebo	3M	-Alveolar osteitis
Torres-Lagares *et al.* (2006)	Spain	Parallel-group RCT	103 (53/50)	34/69	26.78±8.52 (18-64)	0.20% CHX (gel)	Placebo	3M	-Alveolar osteitis
Delilbasi *et al.* (2002)	Turkey	Parallel-group RCT	177 (62/56/59)	82/95	24±nr (nr)	0.20% CHX (rinse)<br>0.20% CHX (rinse) + antibiotics	NaCl	3M	-Alveolar osteitis
Hermesch *et al.* (1998)	USA	Parallel-group RCT	479 (239/240)	101/170	22.30±nr (18-52)	0.12% CHX (rinse)	Placebo	3M	-Alveolar osteitis
Ragno and Szkutnik (1991)	USA	Split-mouth RCT	160 (80/80)	nr	nr	0.12% CHX (rinse)	Placebo	3M	-Alveolar osteitis
Berwick and Lessin (1990)	USA	Parallel-group RCT	77 (39/38)	nr	21.40±nr (16-40)	0.12% CHX (rinse)	NaCl	3M	-Alveolar osteitis
Larsen (1990)	USA	Parallel-group RCT	278 (144/134)	62/78	nr	0.12% CHX (rinse)	Placebo	3M	-Alveolar osteitis
Sanz *et al.* (1989)	USA	Parallel-group RCT	38 (17/21)	nr	nr	0.12% CHX (rinse)	Placebo	Periodontal surgery	-Ephitelization
Fied *et al.* (1988)	United Kingdom	Parallel-group q-RCT	216 (108/108)	223/101	nr	0.20% CHX (rinse)	NaCl	Tooth extraction	-Alveolar osteitis
Krekmanov and Nordenram (1986)	Sweden	Parallel-group q-RCT	110 (37/37/36)	59/51	29±nr (19-59)	0.20% CHX (rinse) + antibiotic<br>0.20% CHX (rinse)<br>0.20% CHX + antibiotics (rinse)	No intervention	3M	-Alveolar osteitis
Tjernberg (1979)	Sweden	Parallel-group q-RCT	60 (30/30)	29/31	26.30±nr (19-38)	0.20% CHX (rinse)	No intervention	3M	-Alveolar osteitis

Abbreviations: n, number; I, intervention group; C, control group; m, males; f, females; y, years; CHX, chlorhexidine; NaCl, saltwater rinse; 3M, third molar; 1M, first molar; SD, standard deviation; nr, not reported; RCT, randomized controlled trial; q-RCT, quasi-randomized controlled trial

3. Meta-analysis on wound healing

3.1 Subgroup meta-analysis by type of intervention

Figure S1. Forest plot graphically representing the stratified meta-analysis by type of intervention on the association between the application of CHX and wound healing.

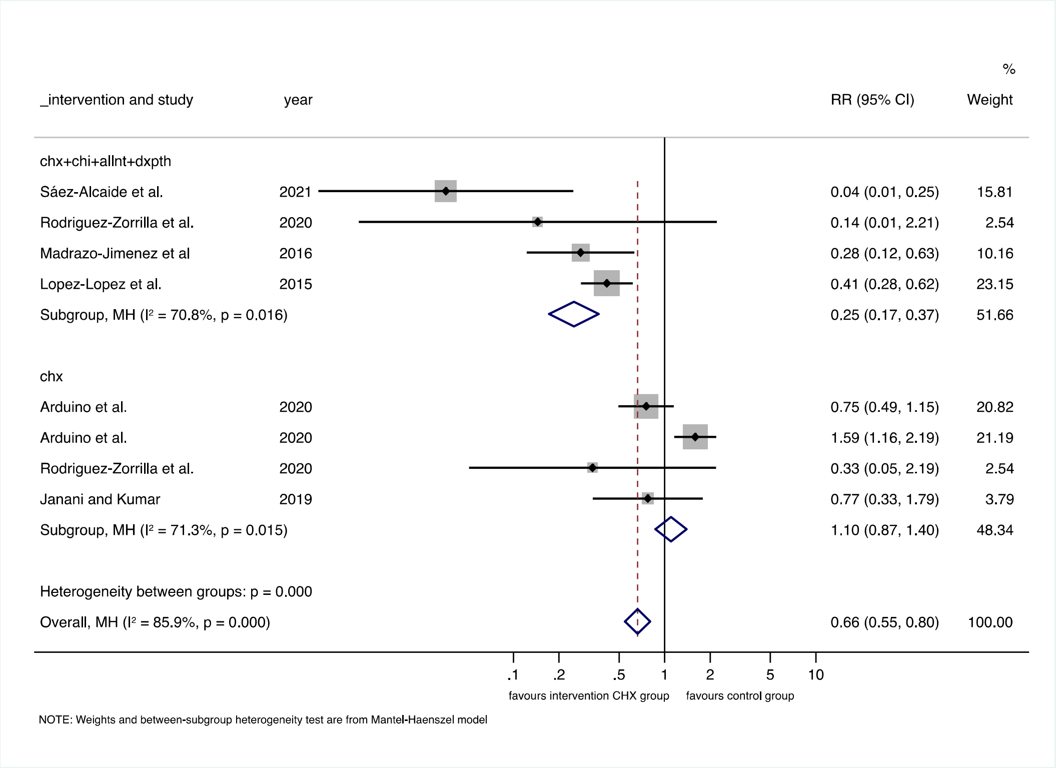

CHX, chlorhexidine; RR, relative risk; CI, confidence intervals. Fixed-effect model, Mantel-Haenszel method. A RR < 1 suggests that the application of CHX is associated with better wound healing. Diamonds indicate the pooled RRs with their corresponding 95% CIs.

3.2 Subgroup meta-analysis by type of vehicle

Figure S2. Forest plot graphically representing the stratified meta-analysis by type of vehicle on the association between the application of CHX and wound healing.

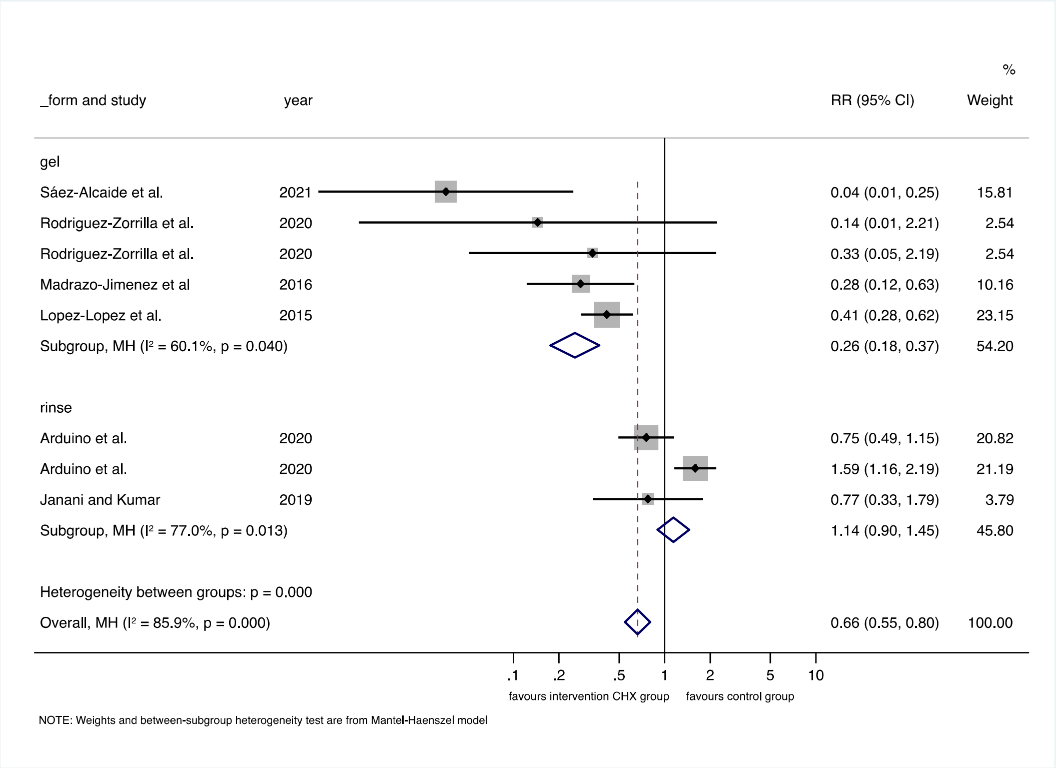

CHX, chlorhexidine; RR, relative risk; CI, confidence intervals. Fixed-effect model, Mantel-Haenszel method. A RR < 1 suggests that the application of CHX is associated with better wound healing. Diamonds indicate the pooled RRs with their corresponding 95% CIs.

3.3 Subgroup meta-analysis by type of concentration

Figure S3. Forest plot graphically representing the stratified meta-analysis by type of concentration on the association between the application of CHX and wound healing.

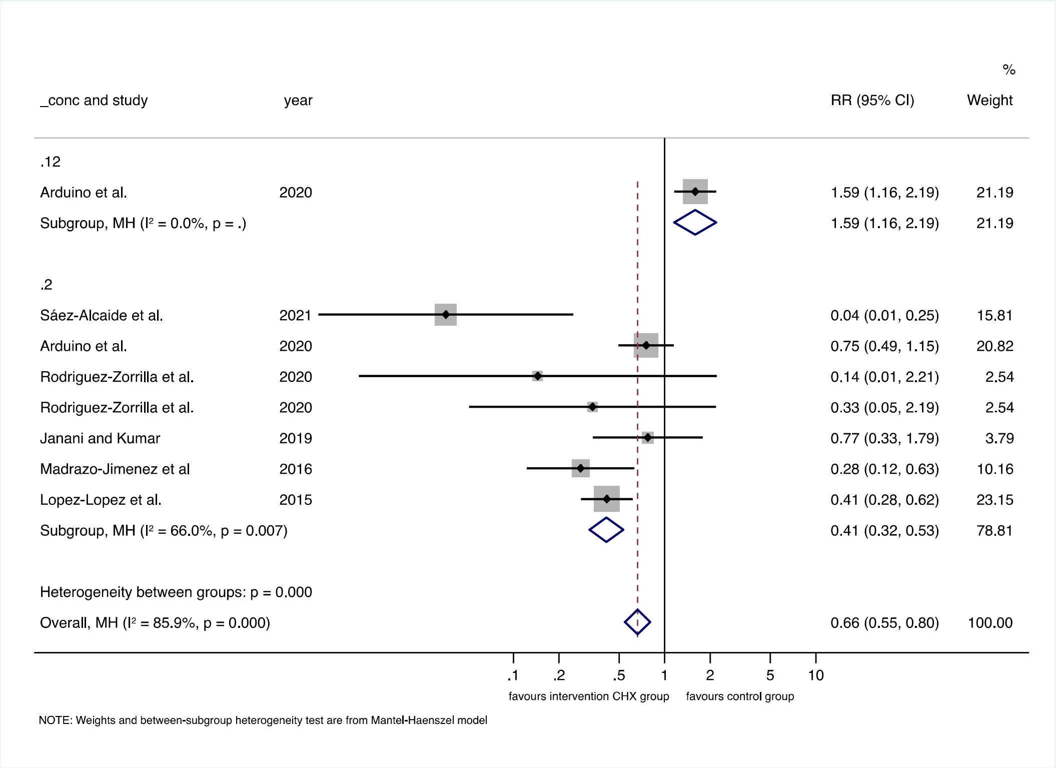

CHX, chlorhexidine; RR, relative risk; CI, confidence intervals. Fixed-effect model, Mantel-Haenszel method. A RR < 1 suggests that the application of CHX is associated with better wound healing. Diamonds indicate the pooled RRs with their corresponding 95% CIs.

3.4 Subgroup meta-analysis by type of oral surgery

Figure S4. Forest plot graphically representing the stratified meta-analysis by type of oral surgery on the association between the application of CHX and wound healing.

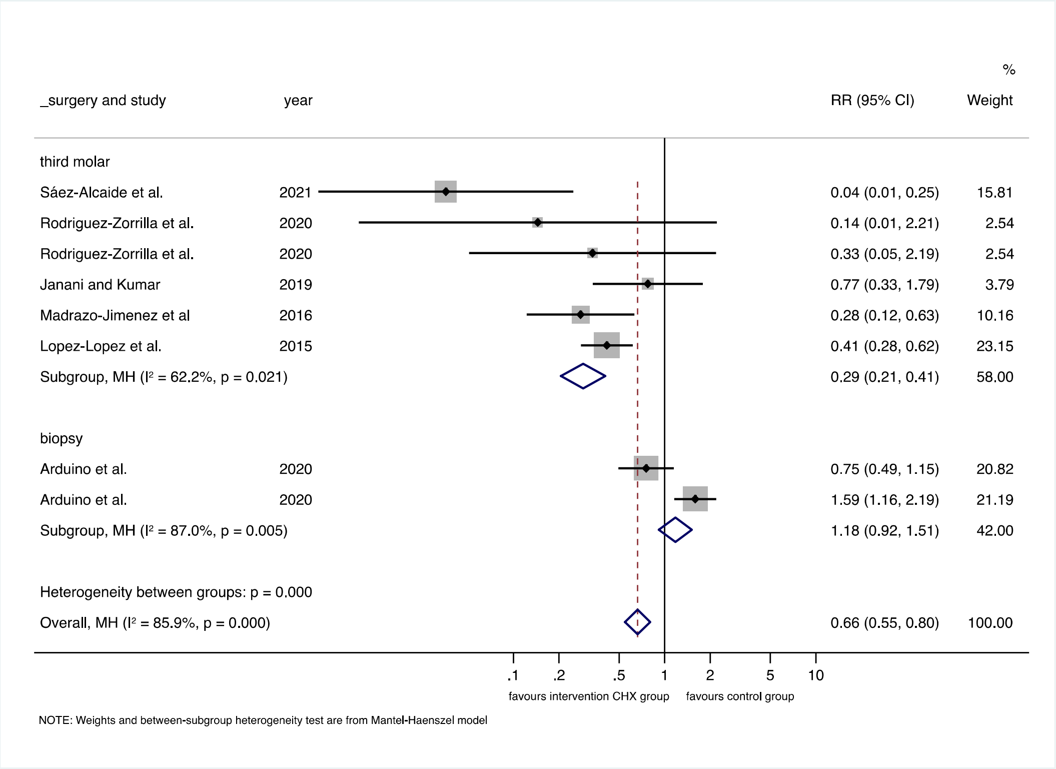

CHX, chlorhexidine; RR, relative risk; CI, confidence intervals. Fixed-effect model, Mantel-Haenszel method. A RR < 1 suggests that the application of CHX is associated with better wound healing. Diamonds indicate the pooled RRs with their corresponding 95% CIs.

3.5 Subgroup meta-analysis by study design

Figure S5. Forest plot graphically representing the stratified meta-analysis by study design on the association between the application of CHX and wound healing.

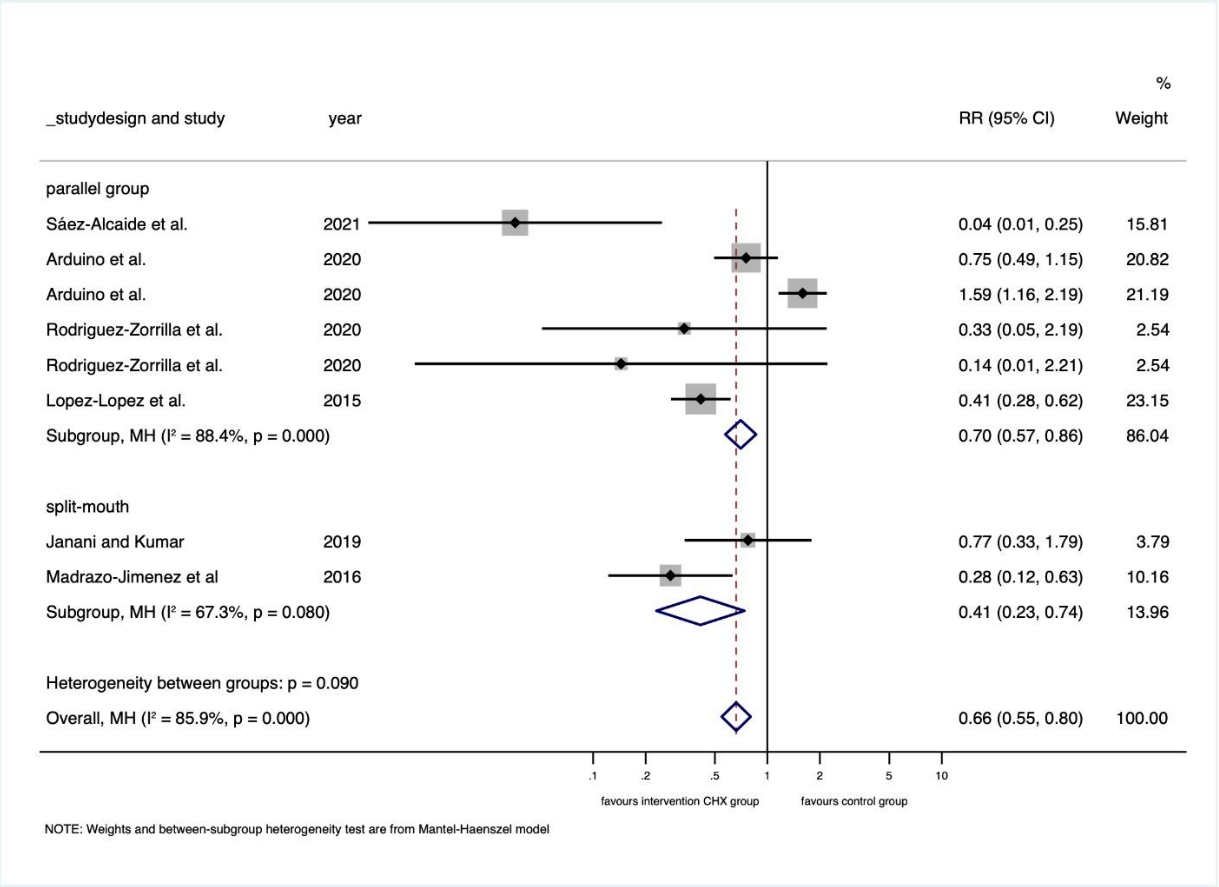

CHX, chlorhexidine; RR, relative risk; CI, confidence intervals. Fixed-effect model, Mantel-Haenszel method. A RR < 1 suggests that the application of CHX is associated with better wound healing. Diamonds indicate the pooled RRs with their corresponding 95% CIs.

3.6 Subgroup meta-analysis by overall RoB

Figure S6. Forest plot graphically representing the stratified meta-analysis by overall RoB on the association between the application of CHX and wound healing.

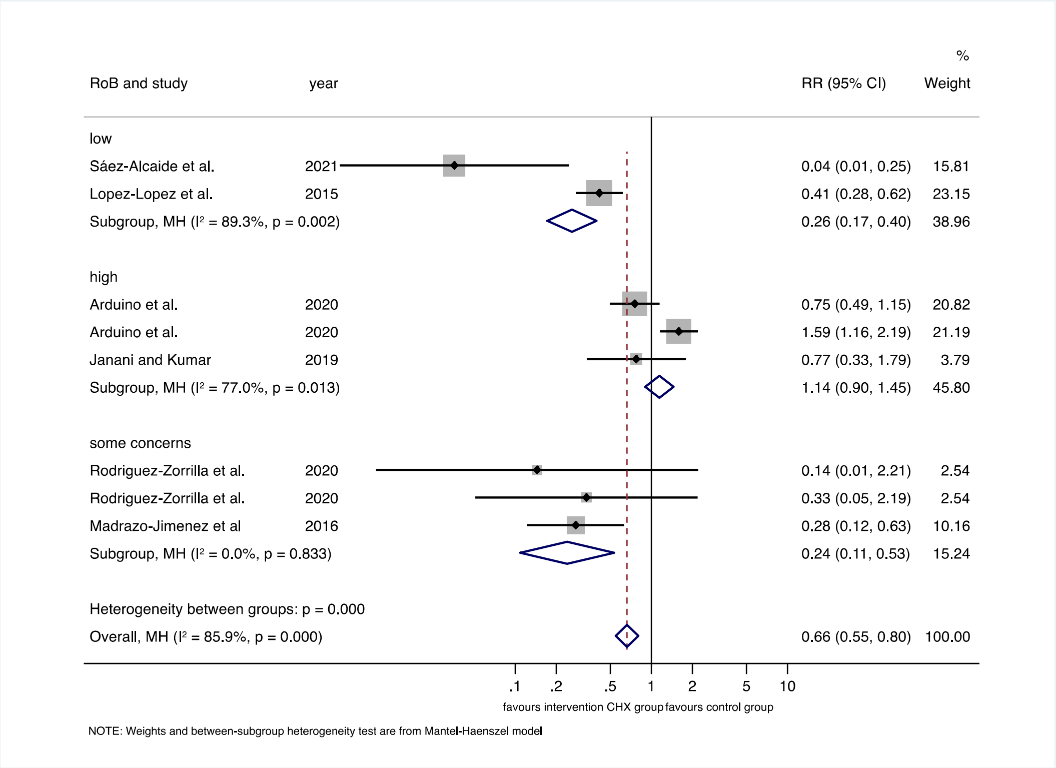

CHX, chlorhexidine; RoB, risk of bias; RR, relative risk; CI, confidence intervals. Fixed-effect model, Mantel-Haenszel method. A RR < 1 suggests that the application of CHX is associated with better wound healing. Diamonds indicate the pooled RRs with their corresponding 95% CIs.

4. Meta-analysis on alveolar osteitis

4.1 Subgroup meta-analysis by type of intervention

Figure S7. Forest plot graphically representing the stratified meta-analysis by type of intervention on the association between the application of CHX and alveolar osteitis.

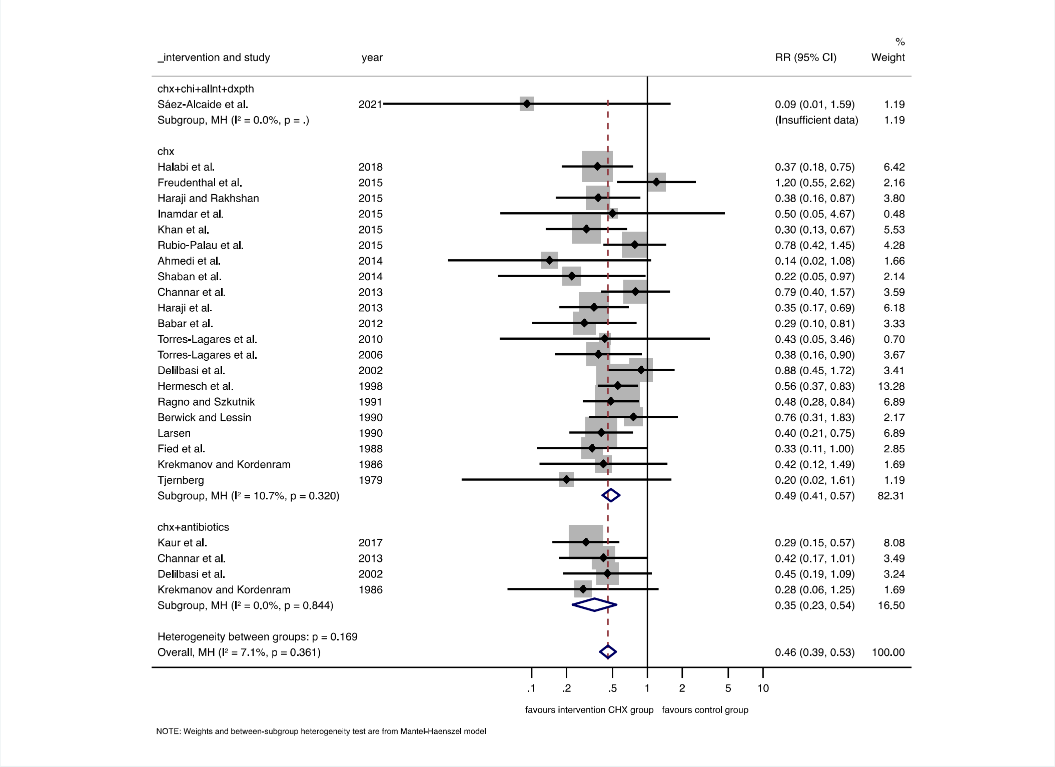

CHX, chlorhexidine; RR, relative risk; CI, confidence intervals. Fixed-effect model, Mantel-Haenszel method. A RR < 1 suggests that the application of CHX is associated with a lower risk of alveolar osteitis. Diamonds indicate the pooled RRs with their corresponding 95% CIs.

4.2 Subgroup meta-analysis by type of vehicle

Figure S8. Forest plot graphically representing the stratified meta-analysis by type of vehicle on the association between the application of CHX and alveolar osteitis.

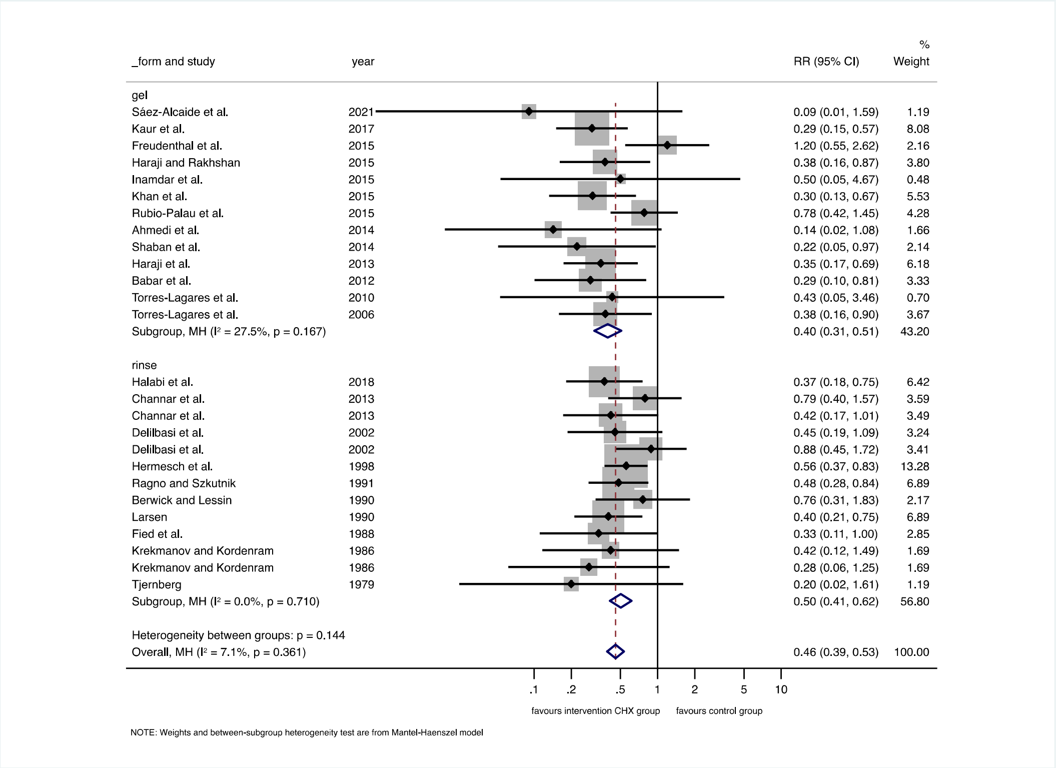

CHX, chlorhexidine; RR, relative risk; CI, confidence intervals. Fixed-effect model, Mantel-Haenszel method. A RR < 1 suggests that the application of CHX is associated with a lower risk of alveolar osteitis. Diamonds indicate the pooled RRs with their corresponding 95% CIs.

4.3 Subgroup meta-analysis by type of concentration

Figure S9. Forest plot graphically representing the stratified meta-analysis by type of concentration on the association between the application of CHX and alveolar osteitis.

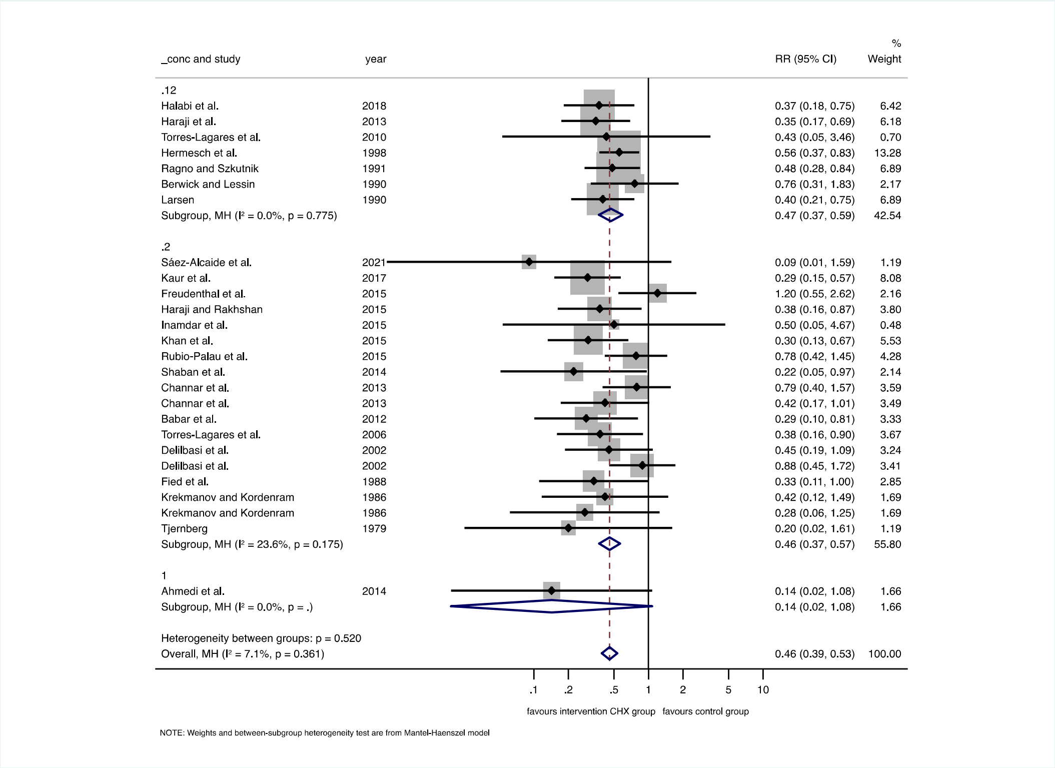

CHX, chlorhexidine; RR, relative risk; CI, confidence intervals. Fixed-effect model, Mantel-Haenszel method. A RR < 1 suggests that the application of CHX is associated with a lower risk of alveolar osteitis. Diamonds indicate the pooled RRs with their corresponding 95% CIs.

4.4 Subgroup meta-analysis by type of oral surgery

Figure S10. Forest plot graphically representing the stratified meta-analysis by type of oral surgery on the association between the application of CHX and alveolar osteitis.

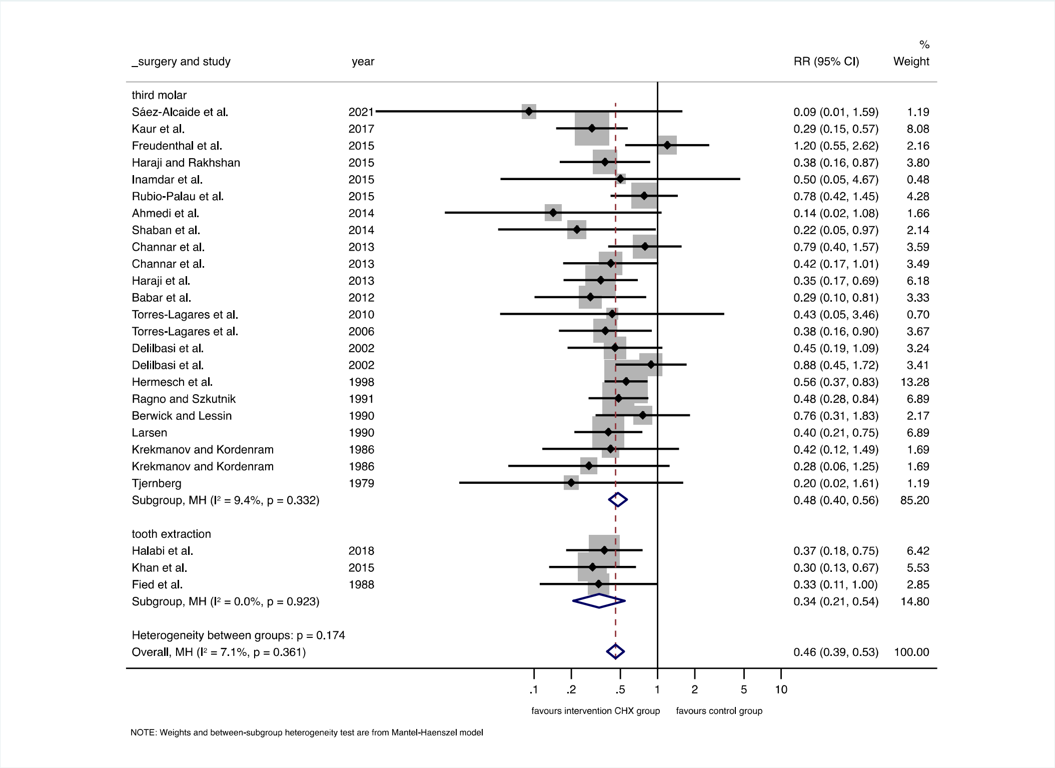

CHX, chlorhexidine; RR, relative risk; CI, confidence intervals. Fixed-effect model, Mantel-Haenszel method. A RR < 1 suggests that the application of CHX is associated with a lower risk of alveolar osteitis. Diamonds indicate the pooled RRs with their corresponding 95% CIs.

4.5 Subgroup meta-analysis by type of study design

Figure S11. Forest plot graphically representing the stratified meta-analysis by type of study design on the association between the application of CHX and alveolar osteitis.

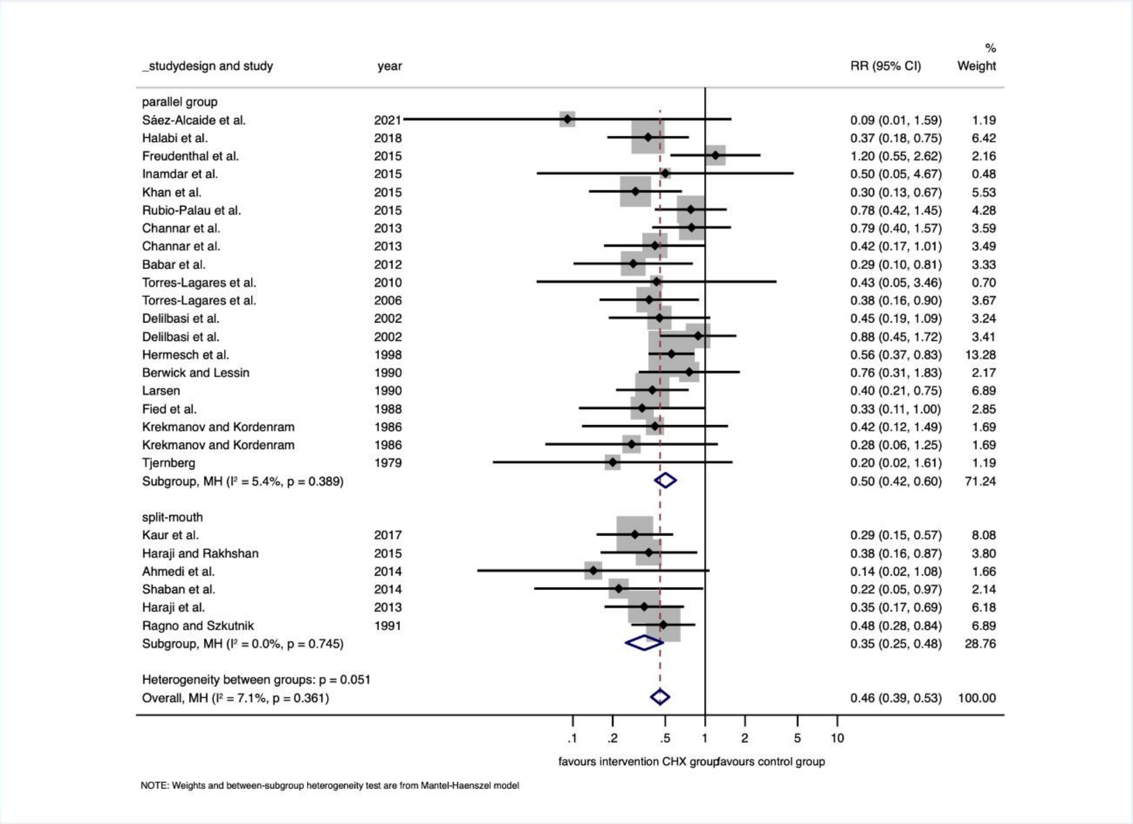

CHX, chlorhexidine; RR, relative risk; CI, confidence intervals. Fixed-effect model, Mantel-Haenszel method. A RR < 1 suggests that the application of CHX is associated with a lower risk of alveolar osteitis. Diamonds indicate the pooled RRs with their corresponding 95% CIs.

4.6 Subgroup meta-analysis by overall RoB

Figure S12. Forest plot graphically representing the stratified meta-analysis by overall RoB on the association between the application of CHX and alveolar osteitis.

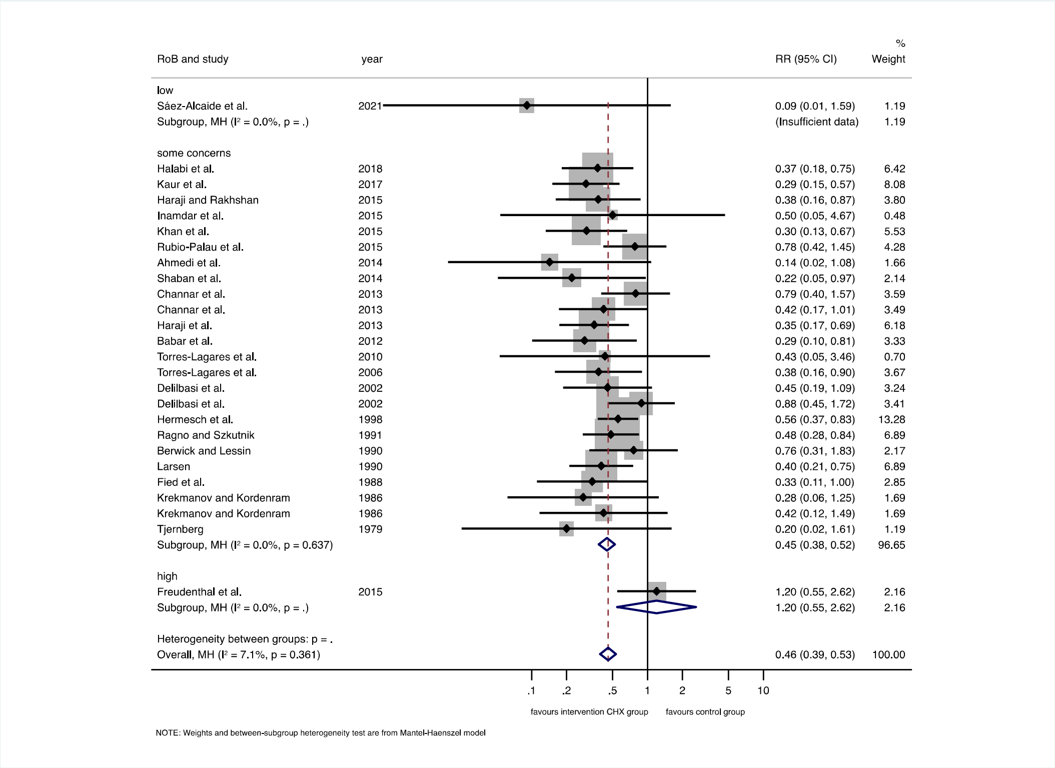

CHX, chlorhexidine; RR, relative risk; CI, confidence intervals. Fixed-effect model, Mantel-Haenszel method. A RR < 1 suggests that the application of CHX is associated with a lower risk of alveolar osteitis. Diamonds indicate the pooled RRs with their corresponding 95% CIs.

5. Meta-analysis on wound erythema

Figure S13. Forest plot graphically representing the meta-analysis on the association between the application of CHX and wound erythema.

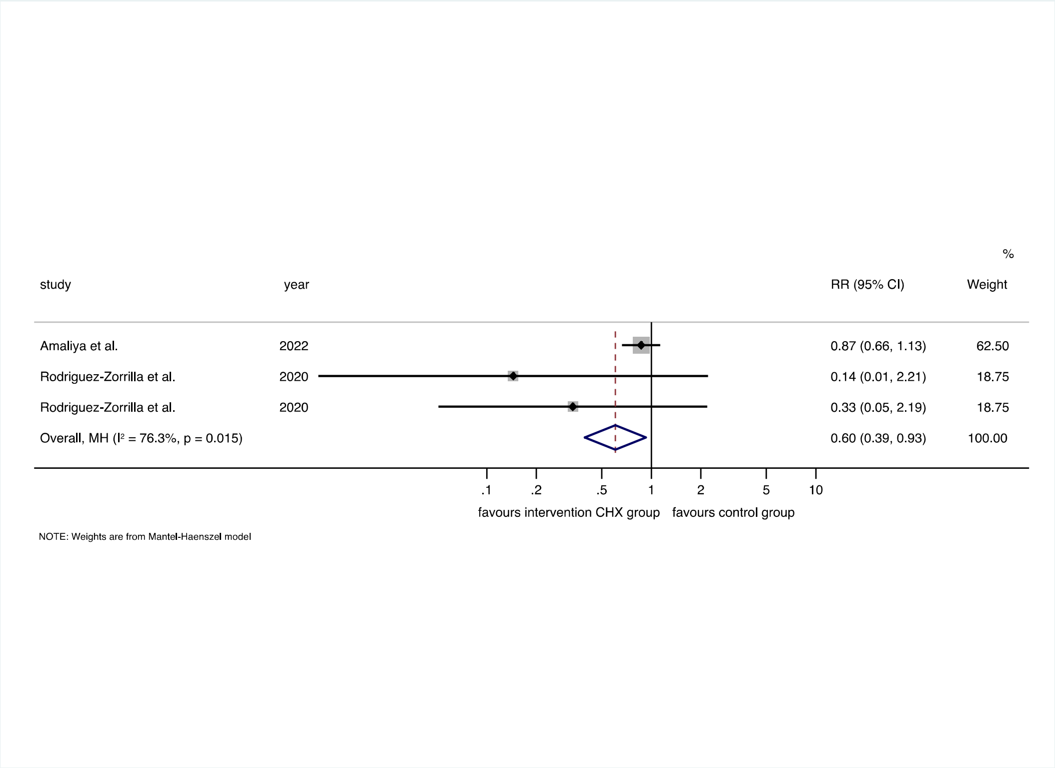

CHX, chlorhexidine; RR, relative risk; CI, confidence intervals. Fixed-effect model, Mantel-Haenszel method. A RR < 1 suggests that the application of CHX is associated with better wound erythema. Diamonds indicate the pooled RRs with their corresponding 95% CIs.

6. Meta-analysis on wound epithelization

Figure S14. Forest plot graphically representing the meta-analysis on the association between the application of CHX and wound epithelization.

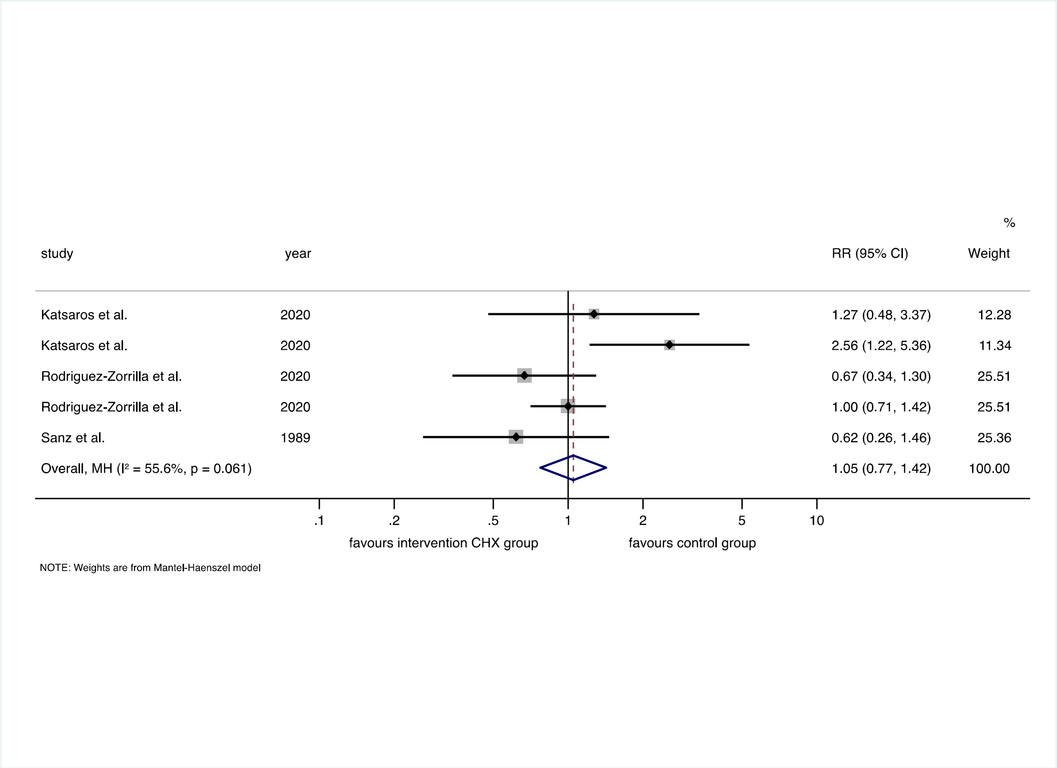

CHX, chlorhexidine; RR, relative risk; CI, confidence intervals. Fixed-effect model, Mantel-Haenszel method. A RR < 1 suggests that the application of CHX is associated with better wound epithelization. Diamonds indicate the pooled RRs with their corresponding 95% CIs.

7. Meta-analysis on pain

7.1 Subgroup meta-analysis by type of intervention

Figure S15. Forest plot graphically representing the stratified meta-analysis by type of intervention on the differences in pain between the CHX group and controls.

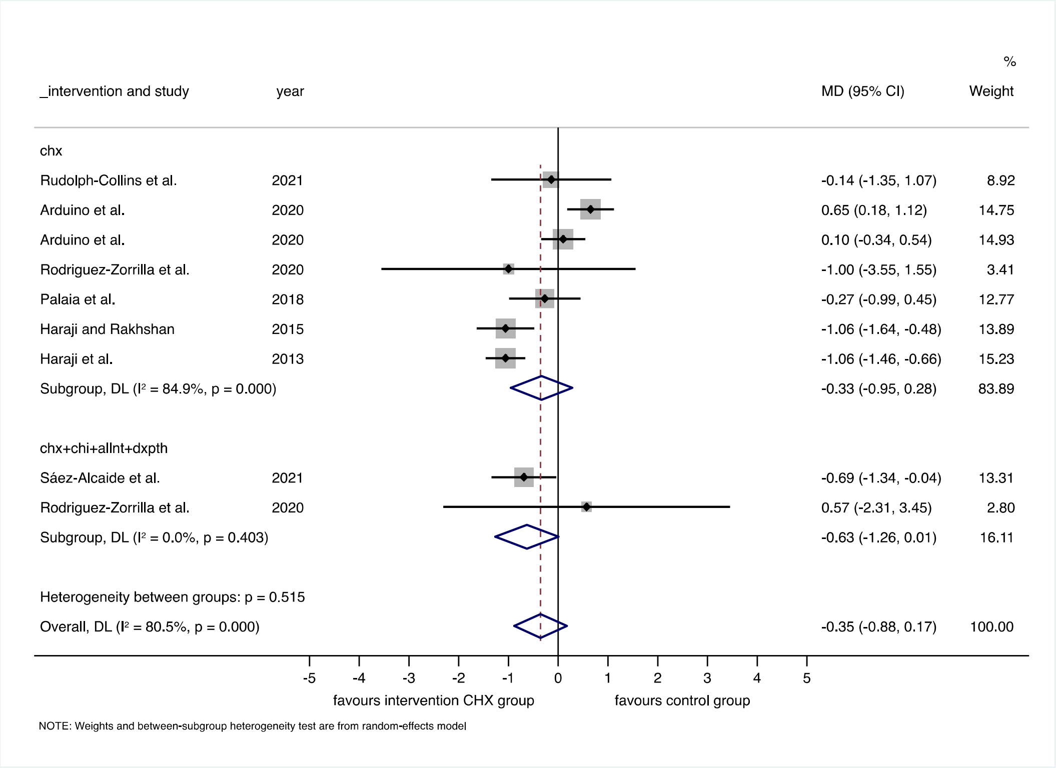

CHX, chlorhexidine; MD, mean difference; CI, confidence intervals. Random-effects model, inverse-variance method. A MD < 0 suggests that pain levels were lower for the CHX group. Diamonds indicate the pooled MD with their corresponding 95% CIs.

7.2 Subgroup meta-analysis by type of vehicle

Figure S16. Forest plot graphically representing the stratified meta-analysis by type of vehicle on the differences in pain between the CHX group and controls.

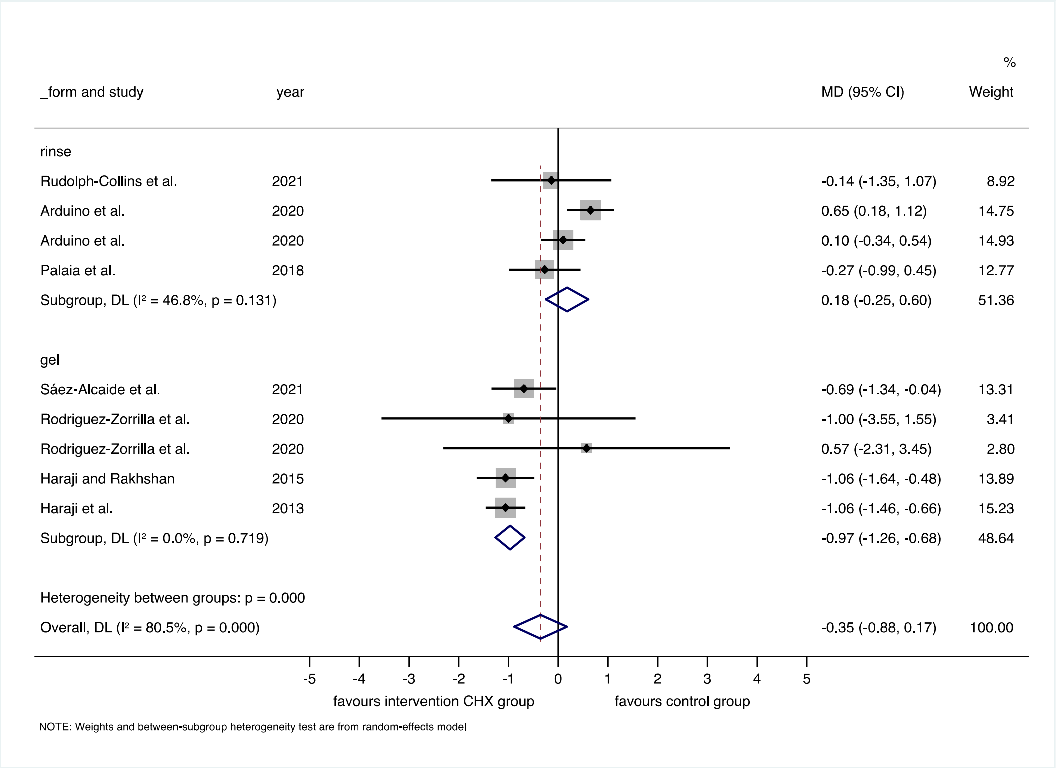

CHX, chlorhexidine; MD, mean difference; CI, confidence intervals. Random-effects model, inverse-variance method. A MD < 0 suggests that pain levels were lower for the CHX is group. Diamonds indicate the pooled MD with their corresponding 95% CIs.

7.3 Subgroup meta-analysis by type of concentration

Figure S17. Forest plot graphically representing the stratified meta-analysis by type of concentration on the differences in pain between the CHX group and controls.

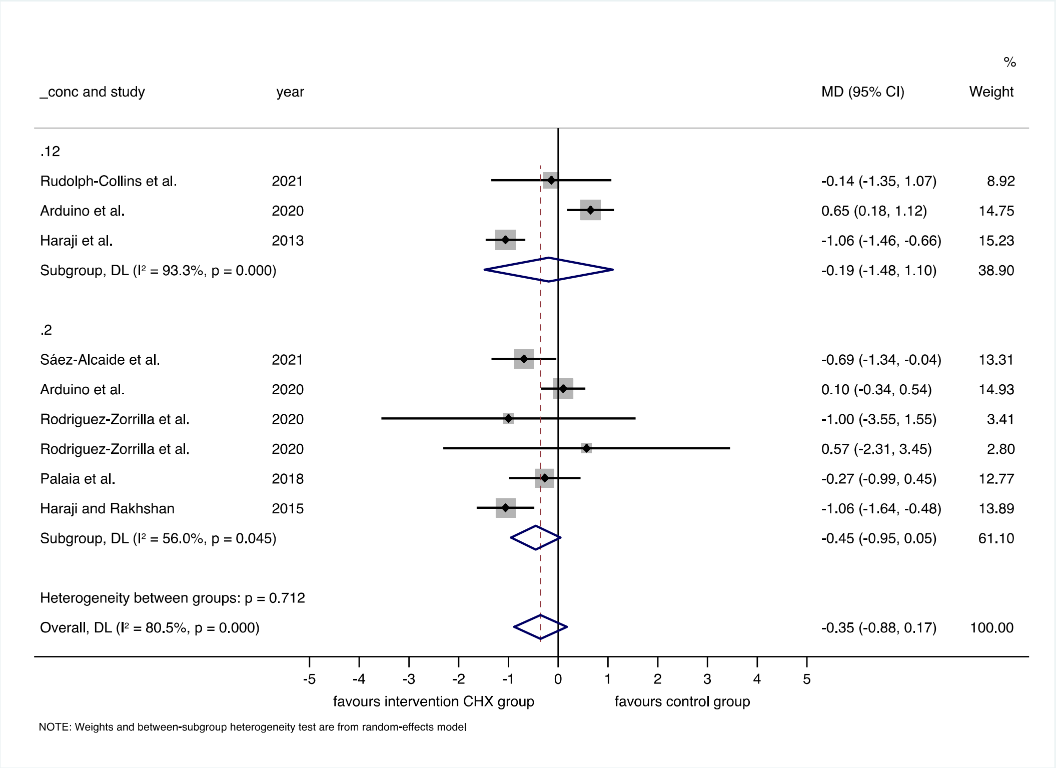

CHX, chlorhexidine; MD, mean difference; CI, confidence intervals. Random-effects model, inverse-variance method. A MD < 0 suggests that pain levels were lower for the CHX is group. Diamonds indicate the pooled MD with their corresponding 95% CIs.

7.4 Subgroup meta-analysis by type of oral surgery

Figure S18. Forest plot graphically representing the stratified meta-analysis by type of oral surgery on the differences in pain between the CHX group and controls.

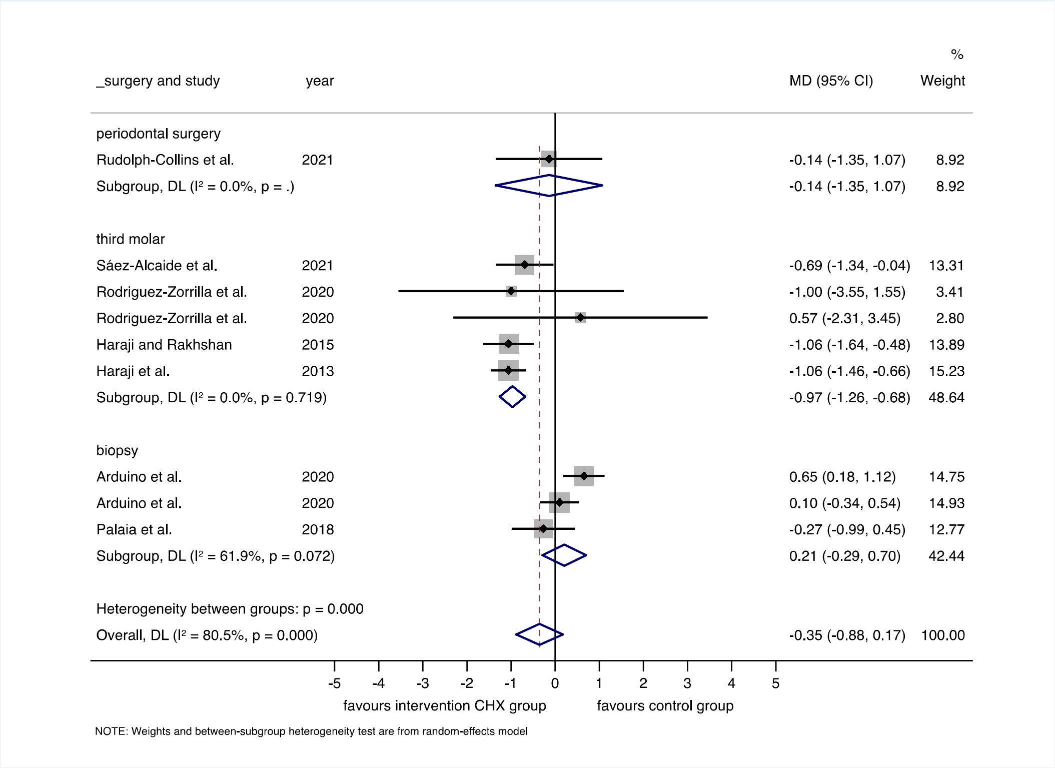

CHX, chlorhexidine; MD, mean difference; CI, confidence intervals. Random-effects model, inverse-variance method. A MD < 0 suggests that pain levels were lower for the CHX is group. Diamonds indicate the pooled MD with their corresponding 95% CIs.

7.5 Subgroup meta-analysis by type of study design

Figure S19. Forest plot graphically representing the stratified meta-analysis by type of study design on the differences in pain between the CHX group and controls.

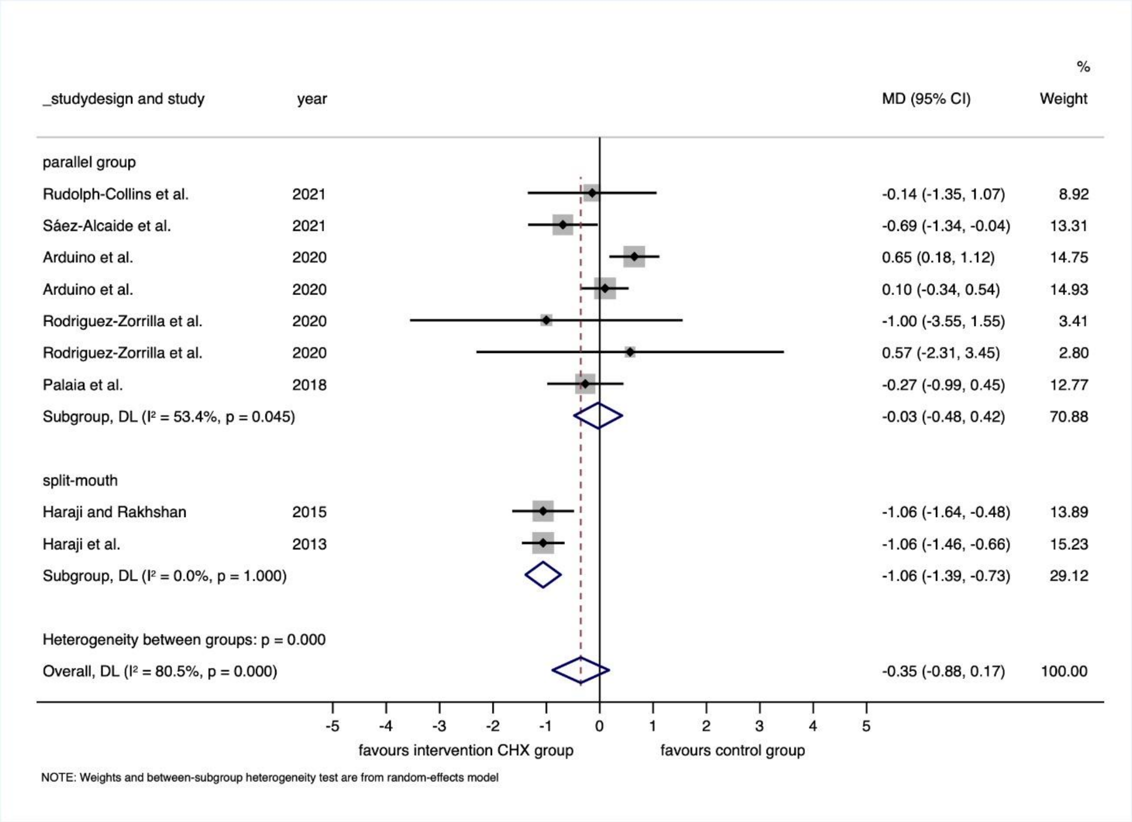

CHX, chlorhexidine; MD, mean difference; CI, confidence intervals. Random-effects model, inverse-variance method. A MD < 0 suggests that pain levels were lower for the CHX is group. Diamonds indicate the pooled MD with their corresponding 95% CIs.

7.6 Subgroup meta-analysis by overall RoB

Figure S20. Forest plot graphically representing the stratified meta-analysis by overall RoB on the differences in pain between the CHX group and controls.

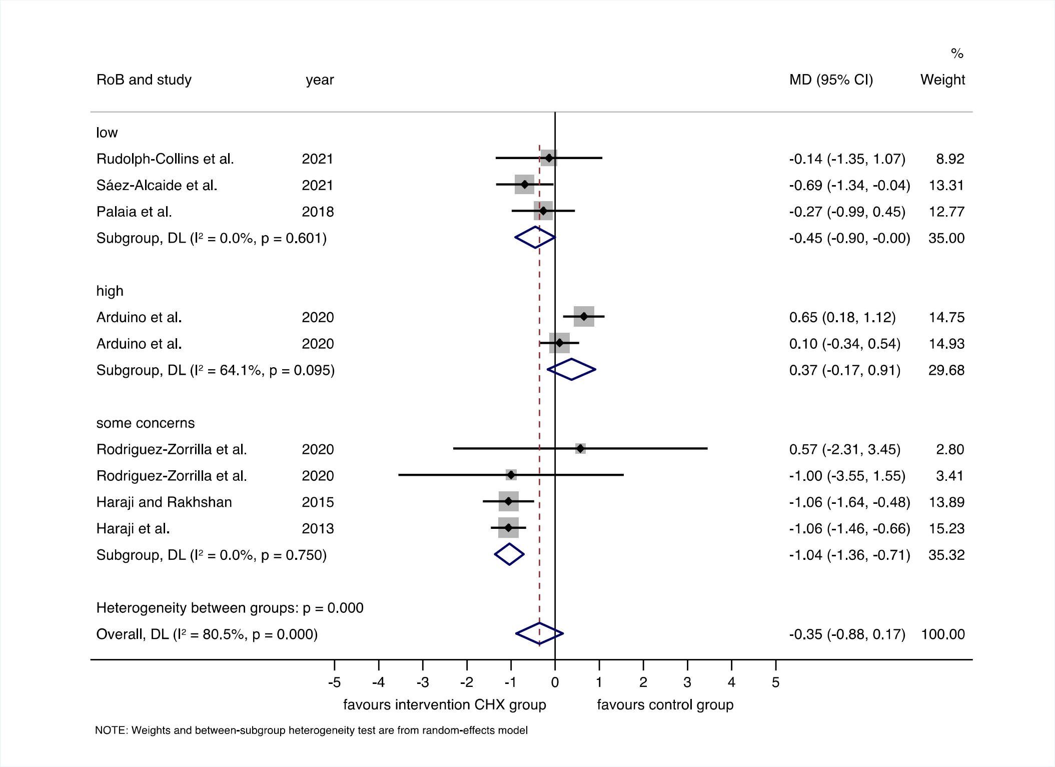

CHX, chlorhexidine; MD, mean difference; CI, confidence intervals. Random-effects model, inverse-variance method. A MD < 0 suggests that pain levels were lower for the CHX is group. Diamonds indicate the pooled MD with their corresponding 95% CIs.

8. Analysis of small-study effects

8.1 Wound healing

Figure S21. A funnel plot of estimated effect sizes against their standard errors, graphically representing the analysis of small-study effects on the association between the application of CHX and wound healing.

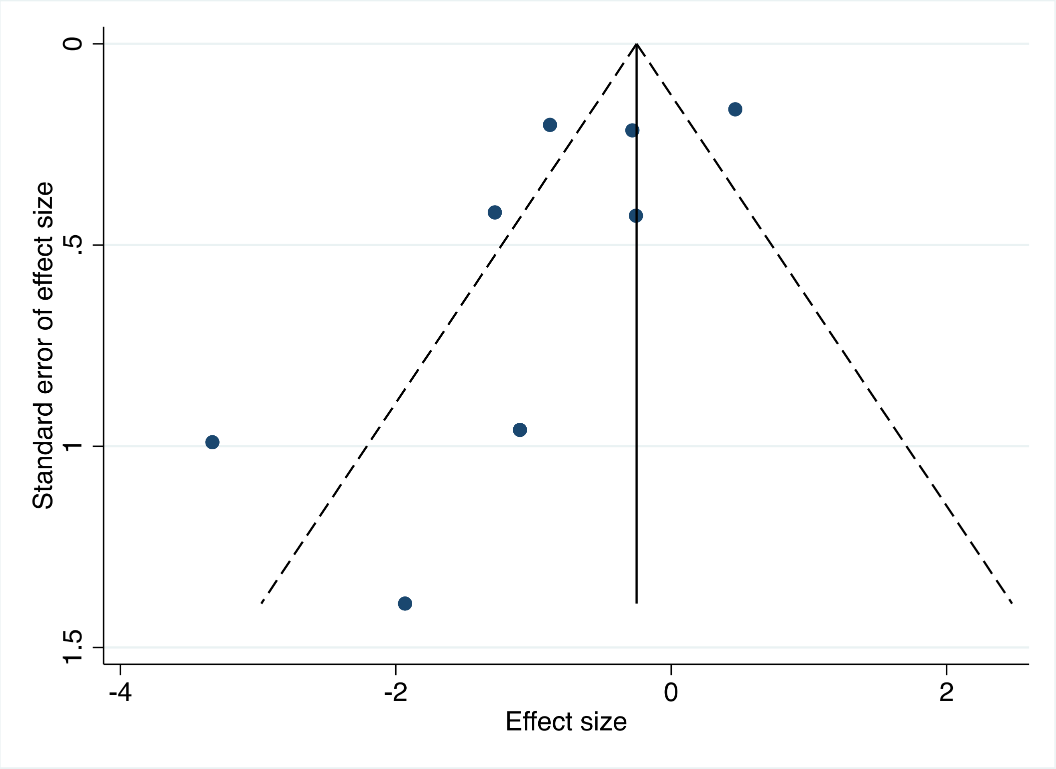

SE, standard error; ES, effect sizes. The black vertical line corresponds to the pooled estimated prevalence. The two diagonal intermittent lines represent the pseudo-95% confidence interval. The blue circles represent the estimates from primary-level studies.

8.2 Alveolar osteitis

Figure S22. A funnel plot of estimated effect sizes against their standard errors, graphically representing the analysis of small-study effects on the association between the application of CHX and alveolar osteitis.

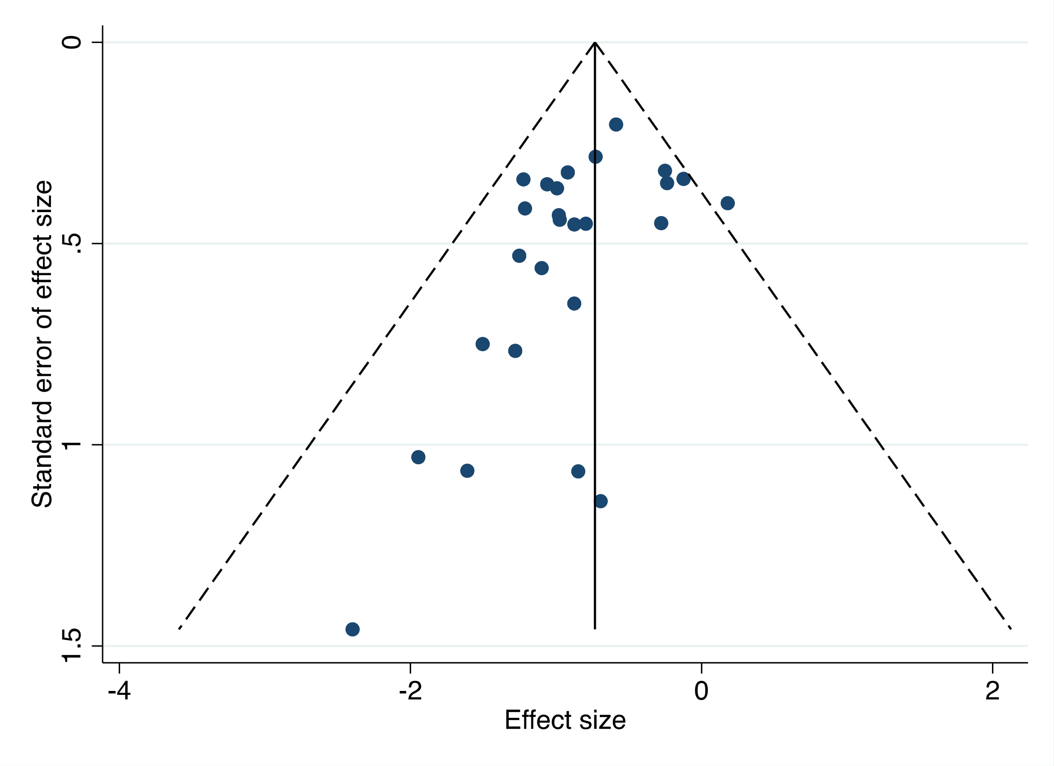

SE, standard error; ES, effect size. The black vertical line corresponds to the pooled estimated prevalence. The two diagonal intermittent lines represent the pseudo-95% confidence interval. The blue circles represent the estimates from primary-level studies.

8.3 Pain

Figure S23. A funnel plot of the differences in pain between CHX group and controls, expressed as mean differences against their standard errors.

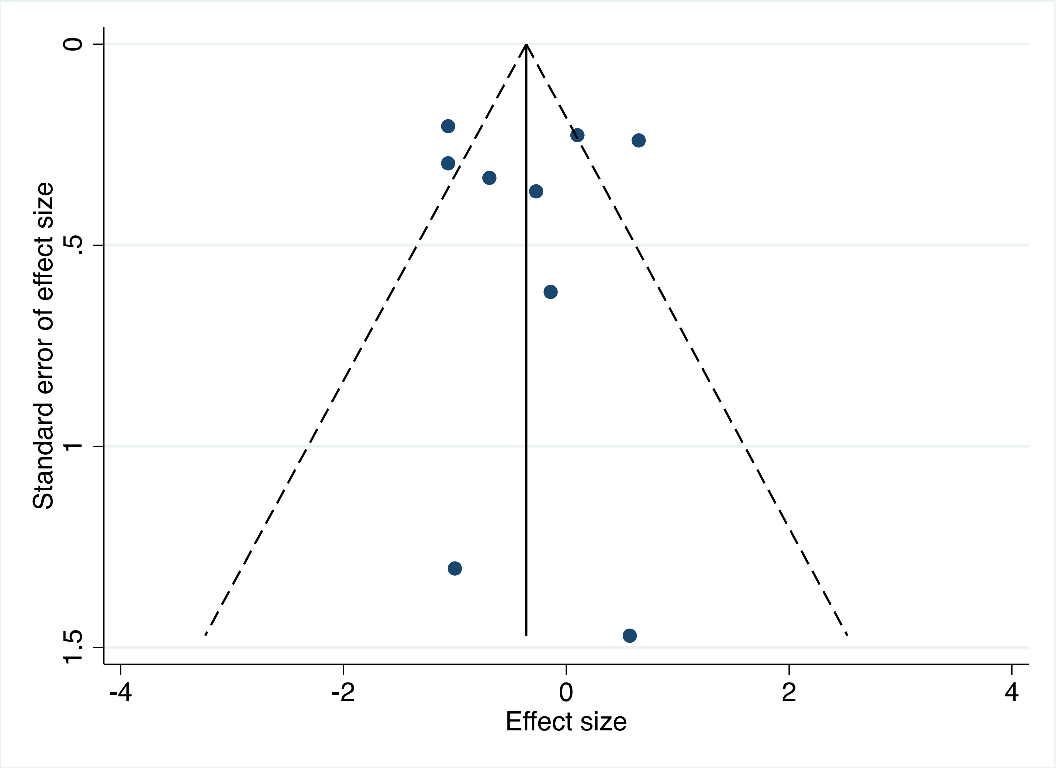

SE, standard error; ES, effect size. The black vertical line corresponds to the pooled estimated prevalence. The two diagonal intermittent lines represent the pseudo-95% confidence interval. The blue circles represent the estimates from primary-level studies.

9. List of full-text articles excluded with reasons

Inappropriate control group (n=27)

- Abu-Mostafa NA, Alqahtani A, Abu-Hasna M, Alhokail A, Aladsani A. A randomized clinical trial compared the effect of intra-alveolar 0.2 % Chlorohexidine bio-adhesive gel versus 0.12% Chlorohexidine rinse in reducing alveolar osteitis following molar teeth extractions. Med Oral Patol Oral Cir Bucal. 2015 Jan 1;20(1):e82-7.

- Ahmedi J, Agani Z, Ademi Abdyli R, Prekazi Loxha M, Hamiti-Krasniqi V, Rexhepi A, Stubljar D. Comparison between ozone and CHX gel application for reduction of pain and incidence of dry socket after lower third molar removal. Clin Exp Dent Res. 2023 Feb;9(1):75-81.

- Almushalbn A, Albassal A, Harfouch M. Comparative Clinical Study Between Chlorhexidine Gel (0.2%) and Hyaluronic Gel (1%) in the Prevention of a Dry Socket After Tooth Extraction for Orthodontic Treatment. Cureus. 2022 Dec 11;14(12):e32391.

- Cho H, David MC, Lynham AJ, Hsu E. Effectiveness of irrigation with chlorhexidine after removal of mandibular third molars: a randomised controlled trial. Br J Oral Maxillofac Surg. 2018 Jan;56(1):54-59.

- Coello-Gómez A, Navarro-Suárez S, Diosdado-Cano JM, Azcárate-Velazquez F, Bargiela-Pérez P, Serrera-Figallo MA, Torres-Lagares D, Gutiérrez-Pérez JL. Postoperative effects on lower third molars of using mouthwashes with super-oxidized solution versus 0.2% chlorhexidine gel: A randomized double-blind trial. Med Oral Patol Oral Cir Bucal. 2018 Nov 1;23(6):e716-e722.

- Cortellini P, Labriola A, Zambelli R, Prato GP, Nieri M, Tonetti MS. Chlorhexidine with an anti discoloration system after periodontal flap surgery: a cross-over, randomized, triple-blind clinical trial. J Clin Periodontol. 2008 Jul;35(7):614-20.

- Divya R, Senthilnathan KP, Kumar MP, Santhosh Murugan P. Effectiveness of herbal and chlorhexidine mouth rinses in the prevention of post-operative complications during third molar surgery. Drug Invention Today 2019 Oct;11:2644-7.

- Eshghpour M, Danaeifar N, Kermani H, Nejat AH. Does intra-alveolar application of chlorhexidine gel in combination with platelet-rich fibrin have an advantage over application of platelet-rich fibrin in decreasing alveolar osteitis after mandibular third molar surgery? A double-blinded randomized clinical trial. J Oral Maxillofac Surg. 2018 May;76(5):939.e1-939.e7.

- Falcao MJ, Mascarenhas P, Noronha S. Application of Bexident Gengivas® vs Bexident Post® – Effects on healing after extraction. Pilot study. Rev Port Estomatol Med Dent Cir Maxilofac. 2017;58(3):192–6.

- Gkatzonis AM, Vassilopoulos SI, Karoussis IK, Kaminari A, Madianos PN, Vrotsos IA. A randomized controlled clinical trial on the effectiveness of three different mouthrinses

(chlorhexidine with or without alcohol and C31G), adjunct to periodontal surgery, in early wound healing. Clin Oral Investig. 2018 Sep;22(7):2581-2591.

- Heitz F, Heitz-Mayfield LJA, Lang NP. Effects of post-surgical cleansing protocols on early plaque control in periodontal and/or periimplant wound healing. J Clin Periodontol. 2004;31:1012-1018.

- Hita-Iglesias P, Torres-Lagares D, Flores-Ruiz R, Magallanes-Abad N, Basallote-Gonzalez M, Gutierrez-Perez JL. Effectiveness of chlorhexidine gel versus chlorhexidine rinse in reducing alveolar osteitis in mandibular third molar surgery. J Oral Maxillofac Surg. 2008 Mar;66(3):441-5.

- Horwitz J, Machtei EE, Zuabi O, Peled M. Amine fluoride/stannous fluoride and chlorhexidine mouthwashes as adjuncts to single-stage dental implants: a comparative study. J Periodontol. 2005 Mar;76(3):334-40.

- Jadhao VA, Rao A, Hande P, Mahajani M, Raktade PP, Gedam R, Acharya V, Tekale PD. The efficiency of three irrigating solutions after surgical removal of impacted mandibular third molars: A cross-sectional study. J Contemp Dent Pract. 2018 Sep 1;19(9):1147-1151.

- Jesudasan JS, Wahab PU, Sekhar MR. Effectiveness of 0.2% chlorhexidine gel and a eugenol-based paste on postoperative alveolar osteitis in patients having third molars extracted: a randomised controlled clinical trial. Br J Oral Maxillofac Surg. 2015 Nov;53(9):826-30.

- Kaposvári I, Körmöczi K, László ZB, Oberna F, Horváth F, Joób-Fancsaly Á. A preoperatív antibiotikus és antiszeptikus kezelés hatása a műtéti úton eltávolított alsó bölcsességfogak sebgyógyulására – prospektív randomizált vizsgálat. Orv Hetil. 2017;158(1):13-9.

- Larsen PE. Alveolar osteitis after surgical removal of impacted mandibular third molars. Identification of the patient at risk. Oral Surg Oral Med Oral Pathol. 1992 Apr;73(4):393-7.

- Laugisch O, Ramseier CA, Salvi GE, Hägi TT, Bürgin W, Eick S, Sculean A. Effects of two different post-surgical protocols including either 0.05% chlorhexidine herbal extract or 0.1% chlorhexidine on post-surgical plaque control, early wound healing and patient acceptance following standard periodontal surgery and implant placement. Clin Oral Investig. 2016 Nov;20(8):2175-2183.

- Lorenzini G, Picciotti M, Giovannardi M, Viviano M. Comparison between two different chlorhexidine-based mouthwashes in oral surgery. Dent Cadmos. 2014;82(9):642-648.

- Metin M, Tek M, Sener I. Comparison of two chlorhexidine rinse protocols on the incidence of alveolar osteitis following the surgical removal of impacted third molars. J Contemp Dent Pract. 2006 May 1;7(2):79-86.

- Mohanty R, Jha C. Randomized study on postoperative intra-alveolar betadine irrigation versus chlorhexidine irrigation on the incidence of occurrence of alveolar osteitis after mandibular third molar surgery. J Maxillofac Oral Surg. 2022 Mar;21(1):163-167.

- Osunde OD, Anyanechi CE, Bassey GO. Prevention of alveolar osteitis after third molar surgery: Comparative study of the effect of warm saline and chlorhexidine mouth rinses. Niger J Clin Pract. 2017 Apr;20(4):470-473.

- Piccione N. Impiego della clorexidina nella terapia di alcune affezioni stomatologiche [Use of chlorhexidine in the therapy of some stomatological diseases]. Minerva Stomatol. 1979 Jul-Sep;28(3):209-14.

- Priyadarshini V, Gajendran PL, Venugopalan S, Nesappan T, Prabhu AR. Comparing the efficacy of two oral therapeutic gels on soft tissue healing following dental implant placement – Pilot study. Int J Pharm Res. 2020 Mar 12; 2599-2608.

- Requena-Calla S, Funes-Rumiche I. Effectiveness of intra-alveolar chlorhexidine gel in reducing dry socket following surgical extraction of lower third molars. A pilot study. J Clin Exp Dent. 2016 Apr 1;8(2):e160-3.

- Rodríguez-Pérez M, Bravo-Pérez M, Sánchez-López JD, Muñoz-Soto E, Romero-Olid MN, Baca-García P. Effectiveness of 1% versus 0.2% chlorhexidine gels in reducing alveolar osteitis from mandibular third molar surgery: a randomized, double-blind clinical trial. Med Oral Patol Oral Cir Bucal. 2013 Jul 1;18(4):e693-700.

- Trombelli L, Simonelli A, Pramstraller M, Guarnelli ME, Fabbri C, Maietti E, Farina R. Clinical efficacy of a chlorhexidine-based mouthrinse containing hyaluronic acid and an antidiscoloration system in patients undergoing flap surgery: A triple-blind, parallel-arm, randomized controlled trial. Int J Dent Hyg. 2018 Nov;16(4):541-552.

<u>No outcomes of interest (n=7)</u>

- Addy M, Dolby AE. The use of chlorhexidine mouthwash compared with a periodontal dressing following the gingivectomy procedure. J Clin Periodontol. 1976 Feb;3(1):59-65.

- Araújo Nobre M, Cintra N, Maló P. Peri-implant maintenance of immediate function implants: a pilot study comparing hyaluronic acid and chlorhexidine. Int J Dent Hyg. 2007 May;5(2):87-94.

- Asboe-Jörgensen V, Attström R, Lang NP, Löe H. Effect of a chlorhexidine dressing on the healing after periodontal surgery. J Periodontol. 1974;45(1):13–7.

- Fotos PG, Koorbusch GF, Sarasin DS, Kist RJ. Evaluation of intra-alveolar chlorhexidine dressings after removal of impacted mandibular third molars. Oral Surg Oral Med Oral Pathol. 1992 Mar;73(3):383-8.

- Langebaek J, Bay L. The effect of chlorhexidine mouthrinse on healing after gingivectomy. Scand J Dent Res. 1976 Jul;84(4):224-8.

- Ozmeric N, Mollaoglu N, Elgun S, Devrim E. Impact of chlorhexidine mouth rinse use on postextraction infection via nitric oxide pathway. Inflamm Res. 2010 Jun;59(6):437-41.

- Sirirat M, Tulananda V. A comparative study of the effect of chlorhexidine mouthwash, periodontal dressing and mechanical tooth cleaning on healing after periodontal flap surgery. J Dent Assoc Thai. 1985 Jan-Dec;35(1-6):13-20.

Inappropriate study design (nonrandomized) (n=2)

- Bonine FL, Larsen PE. Effect of chlorhexidine rinse on the incidence of dry socket in impacted mandibular third molar extraction sites. Oral Surg Oral Med Oral Pathol Oral Radiol Endod. 1995;79(2):154-8.

- Sridhar V, Wali GG, Shyla HN. Evaluation of the perioperative use of 0.2% chlorhexidine gluconate for the prevention of alveolar osteitis after the extraction of impacted mandibular third molars: a clinical study. J Maxillofac Oral Surg. 2011 Jun;10(2):101-11.

Lack of essential data (n = 2)

- Amora-Silva BF, Ribeiro SC, Vieira CL, Mendes FR, Vieira-Neto AE, Abdon APV, Costa FN, Campos AR. Clinical efficacy of new α-bisabolol mouthwashes in postoperative complications of maxillofacial surgeries: a randomized, controlled, triple-blind clinical trial. Clin Oral Investig. 2019 Feb;23(2):577-584.

- Lisbona-González MJ, Muñoz-Soto E, Lisbona-González C, Vallecillo-Rivas M, Diaz-Castro J, Moreno-Fernandez J. Effect of propolis paste and mouthwash formulation on healing after teeth extraction in periodontal disease. plants (basel). 2021 Aug 4;10(8):1603.

Overlapping population (n = 2)

- Haraji A, Khamverdi N, Khanzadealishahi H. The effect of 0.2% chlorhexidine gel in prevention of pain and dry socket following mandibular third molar surgery. J Res Dent Sci 2012; 9(2):63-67

- Torres-Lagares D, Infante-Cossio P, Gutierrez-Perez JL, Romero-Ruiz MM, Garcia-Calderon M, Serrera-Figallo MA. Intra-alveolar chlorhexidine gel for the prevention of dry socket in mandibular third molar surgery. A pilot study. Med Oral Patol Oral Cir Bucal 2006;11:e179-84.

<u>Off topic (n = 1)</u>

- Binahmed A, Stoykewych A, Peterson L. Single preoperative dose versus long-term prophylactic antibiotic regimens in dental implant surgery. Int J Oral Maxillofac Implants. 2005 Jan-Feb;20(1):115-7.
